# Geographic Differences in Genetic Susceptibility to IgA Nephropathy: GWAS Replication Study and Geospatial Risk Analysis

**DOI:** 10.1371/journal.pgen.1002765

**Published:** 2012-06-21

**Authors:** Krzysztof Kiryluk, Yifu Li, Simone Sanna-Cherchi, Mersedeh Rohanizadegan, Hitoshi Suzuki, Frank Eitner, Holly J. Snyder, Murim Choi, Ping Hou, Francesco Scolari, Claudia Izzi, Maddalena Gigante, Loreto Gesualdo, Silvana Savoldi, Antonio Amoroso, Daniele Cusi, Pasquale Zamboli, Bruce A. Julian, Jan Novak, Robert J. Wyatt, Krzysztof Mucha, Markus Perola, Kati Kristiansson, Alexander Viktorin, Patrik K. Magnusson, Gudmar Thorleifsson, Unnur Thorsteinsdottir, Kari Stefansson, Anne Boland, Marie Metzger, Lise Thibaudin, Christoph Wanner, Kitty J. Jager, Shin Goto, Dita Maixnerova, Hussein H. Karnib, Judit Nagy, Ulf Panzer, Jingyuan Xie, Nan Chen, Vladimir Tesar, Ichiei Narita, Francois Berthoux, Jürgen Floege, Benedicte Stengel, Hong Zhang, Richard P. Lifton, Ali G. Gharavi

**Affiliations:** 1 Department of Medicine, College of Physicians and Surgeons, Columbia University, New York, New York, United States of America; 2 Department of Medicine, St. Luke's-Roosevelt Hospital Center, New York, New York, United States of America; 3 Division of Nephrology, Department of Internal Medicine, Juntendo University Faculty of Medicine, Tokyo, Japan; 4 Department of Nephrology, RWTH University of Aachen, Aachen, Germany; 5 Department of Genetics, Howard Hughes Medical Institute, Yale University School of Medicine, New Haven, Connecticut, United States of America; 6 Renal Division, Peking University First Hospital, Peking University Institute of Nephrology, Beijing, China; 7 University of Brescia and Second Division of Nephrology, Montichiari Hospital, Montichiari, Italy; 8 Section of Nephrology, Department of Emergency and Organ Transplantation, University of Bari, Bari, Italy; 9 Nephrology and Dialysis Unit, Ciriè Hospital, Torino, Italy; 10 Department of Genetics, Biology, and Biochemistry, University of Torino, Torino, Italy; 11 Renal Division, DMCO, San Paolo Hospital, School of Medicine, University of Milan, Milan, Italy; 12 Department of Nephrology, Second University of Naples, Naples, Italy; 13 Departments of Microbiology and Medicine, University of Alabama at Birmingham, Birmingham, Alabama, United States of America; 14 Children's Foundation Research Center, Le Bonheur Children's Hospital, Memphis, Tennessee, United States of America; 15 Division of Pediatric Nephrology, University of Tennessee Health Sciences Center, Memphis, Tennessee, United States of America; 16 Department of Immunology, Transplantology, and Internal Medicine, Warsaw Medical University, Warsaw, Poland; 17 Department of Chronic Disease Prevention, National Institute for Health and Welfare, Helsinki, Finland; 18 Institute for Molecular Medicine Finland FIMM, University of Helsinki, Helsinki, Finland; 19 The Estonian Genome Center, University of Tartu, Tartu, Estonia; 20 Department of Medical Epidemiology and Biostatistics, Karolinska Institutet, Stockholm, Sweden; 21 deCODE Genetics, Reykjavik, Iceland; 22 Faculty of Medicine, University of Iceland, Reykjavík, Iceland; 23 Centre National de Génotypage, CEA, Institut de Génomique, Evry, France; 24 INSERM, Centre for Research in Epidemiology and Population Health, Villejuif, France; 25 Nephrology, Dialysis, and Renal Transplantation Department, University North Hospital, Saint Etienne, France; 26 Department of Medicine, Division of Nephrology, University Hospital, Würzburg, Germany; 27 ERA–EDTA Registry, Department of Medical Informatics, Academic Medical Center, University of Amsterdam, Amsterdam, The Netherlands; 28 Division of Clinical Nephrology and Rheumatology, Niigata University, Niigata, Japan; 29 Department of Nephrology, 1st Faculty of Medicine and General University Hospital, Charles University, Prague, Czech Republic; 30 Clinical Research Center and Division of Nephrology, Al Rassoul Al-Aazam Hospital, Beirut, Lebanon; 31 Nephrology Center and 2nd Department of Internal Medicine, Medical Faculty, University of Pécs, Pécs, Hungary; 32 III Medizinische Klinik, Universitätsklinikum Hamburg-Eppendorf, Hamburg, Germany; 33 Department of Nephrology, Ruijin Hospital, Shanghai Jiaotong University School of Medicine, Shanghai, China; University of Oxford, United Kingdom

## Abstract

IgA nephropathy (IgAN), major cause of kidney failure worldwide, is common in Asians, moderately prevalent in Europeans, and rare in Africans. It is not known if these differences represent variation in genes, environment, or ascertainment. In a recent GWAS, we localized five IgAN susceptibility loci on Chr.6p21 (*HLA-DQB1/DRB1, PSMB9/TAP1*, and *DPA1/DPB2* loci), Chr.1q32 (*CFHR3/R1* locus), and Chr.22q12 (*HORMAD2* locus). These IgAN loci are associated with risk of other immune-mediated disorders such as type I diabetes, multiple sclerosis, or inflammatory bowel disease. We tested association of these loci in eight new independent cohorts of Asian, European, and African-American ancestry (N = 4,789), followed by meta-analysis with risk-score modeling in 12 cohorts (N = 10,755) and geospatial analysis in 85 world populations. Four susceptibility loci robustly replicated and all five loci were genome-wide significant in the combined cohort (P = 5×10^−32^–3×10^−10^), with heterogeneity detected only at the *PSMB9/TAP1* locus (I^2^ = 0.60). Conditional analyses identified two new independent risk alleles within the *HLA-DQB1/DRB1* locus, defining multiple risk and protective haplotypes within this interval. We also detected a significant genetic interaction, whereby the odds ratio for the *HORMAD2* protective allele was reversed in homozygotes for a *CFHR3/R1* deletion (P = 2.5×10^−4^). A seven–SNP genetic risk score, which explained 4.7% of overall IgAN risk, increased sharply with Eastward and Northward distance from Africa (r = 0.30, P = 3×10^−128^). This model paralleled the known East–West gradient in disease risk. Moreover, the prediction of a South–North axis was confirmed by registry data showing that the prevalence of IgAN–attributable kidney failure is increased in Northern Europe, similar to multiple sclerosis and type I diabetes. Variation at IgAN susceptibility loci correlates with differences in disease prevalence among world populations. These findings inform genetic, biological, and epidemiological investigations of IgAN and permit cross-comparison with other complex traits that share genetic risk loci and geographic patterns with IgAN.

## Author Summary

IgA nephropathy (IgAN) is the most common cause of kidney failure in Asia, has lower prevalence in Europe, and is very infrequent among populations of African ancestry. A long-standing question in the field is whether these differences represent variation in genes, environment, or ascertainment. In a recent genome-wide association study of 5,966 individuals, we identified five susceptibility loci for this trait. In this paper, we study the largest IgAN case-control cohort reported to date, composed of 10,775 individuals of European, Asian, and African-American ancestry. We confirm that all five loci are significant contributors to disease risk across this multi-ethnic cohort. In addition, we identify two novel independent susceptibility alleles within the HLA-DQB1/DRB1 locus and a new genetic interaction between loci on Chr.1p36 and Chr.22q22. We develop a seven–SNP genetic risk score that explains nearly 5% of variation in disease risk. In geospatial analysis of 85 world populations, the genetic risk score closely parallels worldwide patterns of disease prevalence. The genetic risk score also predicts an unsuspected Northward risk gradient in Europe. This genetic prediction is verified by examination of registry data demonstrating, similarly to other immune-mediated diseases such as multiple sclerosis and type I diabetes, a previously unrecognized increase in IgAN–attributable kidney failure in Northern European countries.

## Introduction

IgA nephropathy (IgAN) is a common kidney disease with a complex genetic determination. This disorder is diagnosed based on detection of mesangial proliferation and glomerular deposits of IgA1. Most frequently, IgAN has a progressing course and 20–50% of cases develop end-stage renal disease (ESRD) within 20 years of follow-up [1]. The disease has been detected among all ethnicities worldwide, but displays a striking geographic variation. It is the most common cause of kidney failure in East Asian countries, has intermediate prevalence in European and US populations but is rarely reported in populations of African ancestry. The diagnosis of IgAN requires a kidney biopsy, complicating accurate determination of heritability and population prevalence of disease. Autopsy and donor biopsy series suggest a prevalence of up to 1.3% in Finland [2] and 3.7% in Japan [3]. Familial aggregation of IgAN has also been recognized throughout the world [4], [5], [6], [7], [8], [9], [10], [11] and up to 14% of cases may be familial [8]. Moreover, family members frequently have aberrant glycosylation of the hinge region of circulating IgA1, a defect with an estimated heritability of 40–50% [12], [13]. These data suggest a strong genetic contribution to disease.

Recently, we have completed a large-scale genome-wide association study (GWAS) involving a cohort of 3,144 sporadic IgAN cases [14]. The discovery phase samples (1,194 cases and 902 controls) were recruited in Beijing, China and were comprised of individuals of Han Chinese ancestry. The most associated SNPs were then followed up in additional cohorts of Han Chinese and Europeans (1,950 cases and 1,920 controls). In the combined analysis, we discovered 5 novel susceptibility loci with consistent effects across individual cohorts. These include 3 distinct intervals in the MHC-II region on chromosome 6p21, with the strongest signal encompassing the HLA *DQB1/DQA1/DRB1* locus (abbreviated as *DQB1/DRB1* hereafter). Imputation of classical alleles suggested that this signal was partially conveyed by a strong protective effect of the DRB1*1501-DQB1*0602 haplotype. The second signal on Chr. 6p21 encompassed a ∼100 Kb region containing *TAP2, TAP1, PSMB8*, and *PSMB9* genes (*TAP2/PSMB9* locus) and the third signal on Chr. 6p21 contained the HLA *DPA1/DPB1/DPB2* genes (*DPA1/DPB2* locus). Independence of these three regions on Chr. 6p21 was demonstrated by their localization within distinct LD blocks as well as genome-wide significant associations after rigorous conditional analyses. We also detected significant association within the *Complement factor H* (*CFH*) gene cluster on Chr. 1q32, where alleles tagging a common deletion in the *CFHR3* and *CFHR1* genes imparted a significant protective effect *(CFHR3/R1* locus). Finally, a fifth signal centered on the *HORMAD2* gene on Chr. 22q12 and containing multiple genes demonstrated significant association with risk of IgAN (*HORMAD2* locus). These five loci individually conferred a moderate risk of disease (OR 1.25–1.59), but together explained 4–5% of the variation in risk across the populations examined.

To follow-up these studies and better assess the risk imparted by susceptibility alleles in diverse populations, we performed a replication study in eight independent case-control cohorts and performed a meta-analysis of all available genetic data including the original GWAS, totaling in 10,755 individuals. The expanded sample size allowed us to formally assess locus heterogeneity, identify new independent risk variants by conditional analyses and search for first-order genetic interactions. Finally, we refined a genetic risk score for IgAN and analyzed differences in the distributions of the IgAN susceptibility alleles among the major world populations.

## Results

### Replication Study

For replication we examined eight cohorts (five European, two East Asian, and one African-American cohort, totaling 2,228 cases and 2,561 controls, described in Table S1). While each individual cohort at best had 40–50% power to replicate original GWAS findings, the combined replication cohort (2,228 cases and 2,561 controls) provided essentially 100% power for replication across the range of allele frequencies and odds ratios initially observed (Table S2).

We genotyped the two top-scoring SNPs for the *CFHR3/R1*, *TAP2/PSMB9*, *DPA1/DPB2*, and *HORMAD2* loci, but four SNPs were included for the *DQB1/DRB1* locus to test for independent alleles at this interval by conditional analysis. After a standard assessment of genotype quality control, we performed association testing within each cohort using the standard Cochrane-Armitage trend test (Table S3). We also tested for heterogeneity of associations and performed a meta-analysis under both fixed and random effects models (Table 1).

**Table 1:** Replication Study Results and Combined Meta-Analysis.

			Replication StudyN = 4,789 across 8 cohorts(2,228 cases/2,561 controls)						Replication and GWASN = 10,755 across 12 cohorts(5,372 cases/5,383 controls)						
			Fixed Effects		Random Effects#				Fixed Effects		Random Effects#				
Chr	Location (kb)	SNP (minor allele)	OR	P-value	OR	P-value	I^2^	Q-test	OR	P-value	OR	P-value	I^2^	Q-test	Annotation
1	194,918	**rs3766404 (C)**	0.78	2.5×10^−4^	0.78	4.2×10^−4^	0%	0.84 (NS)	0.78	7.9×10^−8^	0.78	1.3×10^−7^	6%	0.39 (NS)	*CFHR3/R1* locus
1	194,953	**rs6677604 (A)**	0.78	3.1×10^−5^	0.78	5.5×10^−5^	0%	0.48 (NS)	0.74	2.1×10^−13^	0.74	4.6×10^−13^	21%	0.23 (NS)	
6	32,768	**rs9275224 (A)**	0.75	3.6×10^−11^	0.75	7.1×10^−11^	0%	0.67 (NS)	0.72	8.5×10^−30^	0.72	2.8×10^−29^	0%	0.69 (NS)	*DQB1/DRB1* locus
6	32,778	**rs2856717 (T)**	0.86	1.1×10^−3^	0.86	1.8×10^−3^	0%	0.71 (NS)	0.77	6.6×10^−16^	0.78	7.3×10^−16^	29%	0.16 (NS)	
6	32,779	**rs9275424 (G)**	1.22	5.0×10^−5^	1.22	8.7×10^−5^	19%	0.27 (NS)	1.28	2.6×10^−14^	1.26	4.6×10^−14^	30%	0.14 (NS)	
6	32,789	**rs9275596 (C)**	0.75	5.3×10^−9^	0.75	9.5×10^−9^	0%	0.60 (NS)	0.67	5.0×10^−32^	0.67	3.1×10^−32^	43%	0.05 (NS)	
6	32,917	**rs9357155 (A)**	0.96	5.8×10^−1^	0.97	9.4×10^−2^	54%	0.025*	0.79	1.1×10^−8^	0.87	2.6×10^−11^	70%	1.0×10^−4^ **	*TAP2/PSMB9* locus
6	32,919	**rs2071543 (A)**	0.91	1.7×10^−1^	0.92	1.2×10^−1^	43%	0.08 (NS)	0.78	5.7×10^−10^	0.84	4.0×10^−11^	61%	2.0×10^−3^ **	
6	33,194	**rs1883414 (T)**	0.87	3.1×10^−3^	0.87	5.0×10^−3^	0%	0.96 (NS)	0.82	3.0×10^−10^	0.82	5.9×10^−10^	0%	0.86 (NS)	*DPA1/DPB2* locus
6	33,205	**rs3129269 (T)**	0.89	1.1×10^−2^	0.89	1.7×10^−2^	0%	0.75 (NS)	0.83	2.5×10^−9^	0.83	4.6×10^−9^	0%	0.51 (NS)	
22	28,824	**rs2412971 (A)**	0.81	1.1×10^−6^	0.81	2.1×10^−6^	24%	0.23 (NS)	0.80	4.0×10^−15^	0.80	9.5×10^−15^	12%	0.33 (NS)	*HORMAD2* locus
22	28,859	**rs2412973 (A)**	0.81	6.9×10^−7^	0.81	1.2×10^−6^	29%	0.19 (NS)	0.80	9.9×10^−15^	0.80	2.3×10^−14^	16%	0.29 (NS)	

Combined association results for 12 SNPs representing 5 independent regions that reached genome-wide significance in the original GWAS. The combined effect estimates (per allele odds ratios) in the replication cohorts were all direction-consistent with the ones in the original GWAS cohorts. Significant heterogeneity was noted only for the second HLA locus represented by rs9357155 and rs2071543.

Q-test: P-value for the Cochrane's Q statistic for heterogeneity, NS: heterogeneity test not significant,

*: heterogeneity P<0.05,

**: heterogeneity P<0.01;

I^2^: Heterogeneity Index (0–100%), where <25% corresponds to low, 50%–75% to medium, and >75% to high level of heterogeneity;

OR: Additive (per-allele) Odds Ratio;

# Han and Eskin random effects model.

Four of the five original GWAS loci displayed significant replication with direction-consistent ORs and no heterogeneity comparable to the original findings (Table 1). The strongest replication was at the *DQB1/DRB1* locus and achieved genome-wide significance in the replication cohort (fixed effects OR 0.75, P-value 4×10^−11^). The *CFHFR3/R1* locus on Chr.1q32, the *HORMAD2* locus on Chr.22q12, and the *DPA1/DPB2* locus on Chr.6p21 were also robustly replicated (fixed effects p-values 3×10^−3^–7×10^−7^), with minimal between-cohort heterogeneity (I^2^<25%). Accordingly, when combined with the four cohorts studied in the original GWAS, these four loci provided highly significant evidence of association (fixed effects p-values 3×10^−10^–5×10^−32^).

In contrast, the *TAP2/PSMB9* locus on Chr. 6p21 displayed direction-consistent replication only in the Italian, German, Czech, and Japanese cohort but the full replication cohort did not support this association (Table 1, Table S3). However, when combined with the four cohorts from the original GWAS, this locus remained genome-wide significant (fixed effects p-values 1×10^−8^ and 6×10^−10^ for rs9357155 and rs2071543, respectively, Table 1). As expected, I^2^ and Q-tests provided evidence of heterogeneity and random effects meta-analysis, which explicitly models heterogeneity, was 1–3 orders of magnitude more significant than fixed effect meta-analysis at this interval (e.g. random effects p-value 3×10^−11^, I^2^ = 61% for rs9357155; Table 1). The heterogeneity was not attributable to differences in ethnicity or cohort size as the association results varied within Asian and European cohorts of differing size (Table S3).

### Conditional Analysis Reveals New Independent Risk Alleles within the *HLA-DQB1/DRB1* Locus

The top signals in the original GWAS, represented by rs9275596 and located within the *DQB1/DRB1* locus, were mediated by a very strong protective effect of the DRB1*1501-DQB1*602 haplotype [14]. However, the SNPs in this interval are in incomplete LD and conditional analyses in our GWAS [14] and in an independent study of Europeans [15] had indicated that additional independent haplotypes also contributed to the signal. Therefore, taking advantage of our expanded cohort size, we examined additional SNPs that were in partial LD with rs9275596 to detect potentially independent effects (rs9275224, rs2856717 and rs9275424, which had an r^2^ of 0.09 to 0.7 with rs9275596, Table S4).

After mutually conditioning each SNP on the remaining SNPs, three of the four SNPs in the *DQB1/DRB1* region exhibited a genome-wide significant independent effect (rs9275596, rs9275224 and rs2856717, conditioned p-vales<5×10^−8^, Table 2). Interestingly, the conditioned effect of the minor allele of rs2856717 was reversed compared to the crude effect estimate, suggesting that the adjustment for LD structure has uncovered a risk haplotype in this region (conditioned OR 1.61, p = 2×10^−10^).

**Table 2:** Conditional analysis of the *HLA-DQB1, HLA-DQA1, HLA-DRB1* locus.

	Replication StudyN = 4,789 across 8 cohorts(2,228 cases/2,561 controls)				Replication and GWASN = 10,755 across 12 cohorts(5,372 cases/5,383 controls)				
	UNADJUSTED		CONDITIONED		UNADJUSTED		CONDITIONED		
	OR	P-value	OR	P-value	OR	P-value	OR	P-value	CONDITIONING SNPs
**rs9275224**	0.75	4×10^−11^	0.71	2×10^−6^	0.72	9×10^−30^	0.75	7×10^−10^	rs2856717, rs9275424, rs9275596
**rs2856717**	0.86	1×10^−3^	1.72	1×10^−6^	0.77	7×10^−16^	1.61	2×10^−10^	rs9275224, rs9275424, rs9275596
**rs9275424**	1.22	5×10^−5^	1.06	3×10^−1^	1.28	3×10^−14^	1.11	7×10^−3^	rs9275224, rs2856717, rs9275596
**rs9275596**	0.75	5×10^−9^	0.64	2×10^−6^	0.67	5×10^−32^	0.58	3×10^−16^	rs9275224, rs2856717, rs9275424

The above data indicated that there are multiple risk haplotypes within the *DQB1/DRB1* locus. To better define these findings, we next phased four-SNP haplotypes at this locus and tested associations with disease (Table 3). We confirmed a very strong protective effect of the ATAC haplotype (freq. 0.21) which, based on our previous imputation analysis, carries the DRB1*1501/DQB1*602 classical alleles. In addition, we defined a new protective haplotype (ACAT, freq. 0.13) and a new risk haplotype (ATAT, freq. 0.05). The ATAC protective haplotype and the ATAT risk haplotype differ only by the rs9275596-C/T allele, explaining the reversal of OR for the rs2856717-T allele after conditioning for rs9275596 (Table 3). Additionally, the GCGT risk haplotype, tagged by the rs9275424-G allele, exhibited a weaker protective effect. These results were supported by both Asian and European cohorts (Table S5). Further support is provided by the global haplotype association test, which achieved a p-value of 3×10^−43^. Based on these analyses, we concluded that there are at least three independent haplotypes conferring risk of IgAN within this region.

**Table 3:** Haplotype analysis of rs9275224, rs2856717, rs9275424, and rs9275596 at the HLA-DQB1/DRB1 locus.

All Cohorts: N = 10,755 (5,372 cases/5,383 controls)						
	Freq. Overall	Freq. Cases	Freq. Controls	OR	95%CI	P-global
**GCAT**	0.352	0.365	0.338	-reference-	-reference-	3×10^−43^
**ATAC**	0.213	0.180	0.245	0.69	0.64–0.74	
**ACAT**	0.130	0.119	0.141	0.78	0.71–0.85	
**ATAT**	0.050	0.058	0.043	1.25	1.10–1.42	
**GCGT**	0.246	0.270	0.222	1.12	1.04–1.20	

The most common haplotype of 4 major alleles (GCAT) is used as a reference to derive odds ratios for all other haplotypes. Only common haplotypes (frequency >1%) are tested for association.

Nonetheless, these 3 independent haplotypes in *DQB1/DRB1* locus still did not explain associations in other Chr. 6p21 regions (*TAP2/PSMB9* and *DPA1/DPB2* loci, respectively represented by rs9357155 and rs1883414), and a fully adjusted model that included all independently associated SNPs continued to support the original GWAS findings of three discrete genome-wide significant intervals on Chr. 6p21 (Table 4).

**Table 4:** The best predictive model for IgAN based on all the genotyped SNPs and their pairwise interaction terms.

	Best Predictive Model		
Predictor (Reference Allele)	Coeficient (β)	OR (95%CI)	P-value	Chr.	Annotation of Genes in the Region
**rs6677604 (A)**	−0.49371	0.61 (0.53–0.71)	2.2×10^−11^	1q32	*CFH, CFHR1, CFHR3*
**rs9275224 (A)**	−0.31307	0.73 (0.67–0.80)	2.5×10^−11^	6p21	*HLA-DQB1, -DQA1, -DRB1 (variant 1)*
**rs2856717 (T)**	0.42265	1.53 (1.31–1.78)	8.2×10^−8^	6p21	*HLA-DQB1, -DQA1, -DRB1 (variant 2)*
**rs9275596 (C)**	−0.51157	0.60 (0.52–0.69)	5.9×10^−13^	6p21	*HLA-DQB1, -DQA1, -DRB1 (variant 3)*
**rs9357155 (A)**	−0.28621	0.75 (0.69–0.82)	3.8×10^−10^	6p21	*HLA-DOB, PSMB8, PSMB9, TAP1, TAP2*
**rs1883414 (T)**	−0.1805	0.83 (0.78–0.90)	4.8×10^−7^	6p21	*HLA-DPB2, -DPB1, -DPA1*
**rs2412971 (A)**	−0.28592	0.75 (0.70–0.81)	2.3×10^−15^	22q12	*HORMAD2, MTMR3, LIF, OSM, GATSL3, SF3A1*
rs6677604 (A)* rs2412971 (A)	0.23171	1.26 (1.12–1.43)	2.2×10^−4^	–	*1q32 by 22q12 interaction term*

This model represents the solution of a stepwise logistic regression algorithm (BIC-based stepwise model selection). The coefficients from this model are used to refine the risk score for IgAN.

### First-Order Interaction Screen Reveals Significant Interaction between *CFHR3/R1* and *HORMAD2* Loci

**Figure 1:**
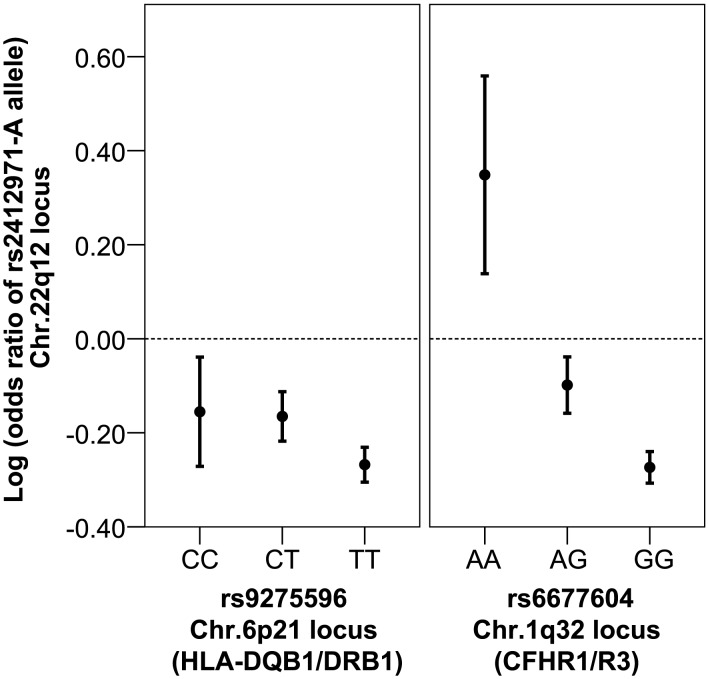
Multiplicative interaction between Chr. 22q12 (rs2412971) and Chr. 1q32 (rs6677604) loci. The allelic effects of rs2412971-A by genotype class of rs9275596 (top signal in the HLA, no interaction) and rs6677604 (top signal in at *CFHR1/R3* locus on Chr. 1q32, significant interaction). The protective effect of rs2412971-A allele is reversed in homozygotes for the rs6677604-A allele, which tags a deletion in *CFHR3/R1*. The allelic effects are expressed on the log-odds scale and correspond to beta coefficients of the logistic regression model. Error bars correspond to 95% confidence intervals.

We tested the possibility of interaction between the 7 risk-contributing SNPs and therefore tested for all possible pairwise interactions (Table S6). We detected strong evidence for a multiplicative interaction (defined as departure from additivity on the log-odds scale) between the *CFHR3/R1* (rs6677604) and the *HORMAD2* loci (rs2412971). In this interaction, the rs2412971-A allele has a strong and consistent protective effect among all genotypic subgroups, but its effects are reversed among homozygotes for the rs6677604-A allele, which closely tags a *CFHR3/R1* deletion (Figure 1, Table S6). The significance of this interaction (p = 2.5×10^−4^) exceeds a Bonferroni-corrected threshold for 21 tests, and is most discernable among the European cohorts (p = 1.4×10^−3^), where both SNPs have higher minor allele frequencies. The 4-df genotypic interaction test was also significant for these two loci (p = 6.4×10^−3^), but the 1-df multiplicative interaction model provided a better fit.

### Improved Prediction of Genetic Risk with a Refined Risk Score

The original IgAN risk score model was based on the genotypes of the top scoring SNPs at the 5 independent loci discovered in the GWAS [14]. We refined this risk score by incorporating the newly discovered independent effects of rs9275224 and rs2856717 and the interaction between the *CFHR3/R1* and the *HORMAD2* loci. A stepwise regression algorithm in the entire cohort defined a new risk score that retained the 7 SNPs exhibiting an independent effect as well as the rs6677604* rs2412971 interaction term (Table 4). When compared with the original GWAS model, the newly refined score was more strongly associated with disease risk and explained a greater proportion of the disease variance in both the replication and the original GWAS dataset (Table 5). Moreover, the refined risk score was a highly significant predictor of disease in each individual replication cohort (Table S7). In all datasets combined, the new risk score explained 4.7% in disease variance and was 13 orders of magnitude more significant than the original score. In this model, one standard deviation increase in the score was associated with nearly 50% increase in the odds of disease (OR = 1.47, 95% CI: 1.42–1.54, P = 1.2×10^−72^). This translates into nearly a 5-fold increase in risk between individuals from the opposing extremes of the risk score distribution (with tails defined by ≥2 standard deviations from the mean).

**Table 5:** The comparison of the original and the newly refined IgAN risk score.

		Original Risk Score				Newly Refined Risk Score			
Cohort:	N#	R^2^ *	C**	OR***	P-value****	R^2^ *	C**	OR***	P-value****
**Original GWAS Cohorts**	5,631	5.0%	0.61	1.51	3.1×10^−46^	5.7%	0.62	1.56	4.1×10^−52^
**Replication Cohorts**	4,422	2.2%	0.58	1.29	5.4×10^−17^	3.2%	0.59	1.36	3.3×10^−24^
**Asian Cohorts Combined**	4,582	4.5%	0.60	1.53	3.0×10^−34^	5.0%	0.61	1.52	2.6×10^−38^
**European Cohorts Combined**	5,386	2.6%	0.58	1.34	3.7×10^−24^	3.6%	0.59	1.42	6.7×10^−33^
**All Cohorts Combined**	10,053	3.8%	0.60	1.42	6.2×10^−63^	4.7%	0.61	1.47	1.2×10^−76^

The expanded version of this table can be found in supplemental material (Table S7).

# Number of analyzed individuals with 100% non-missing genotypes across all 7 scored loci.

*: 2: Nagelkerke R square (expressed as percentage).

**: C-statistic: area under the ROC curve.

***: Odds ratio per one standard deviation of the standardized risk score.

****: Wald's test for risk score as a quantitative predictor of disease status.

### Geospacial Modeling of Genetic Risk Reveals New Geographic Patterns in Disease Prevalence

**Figure 2:**
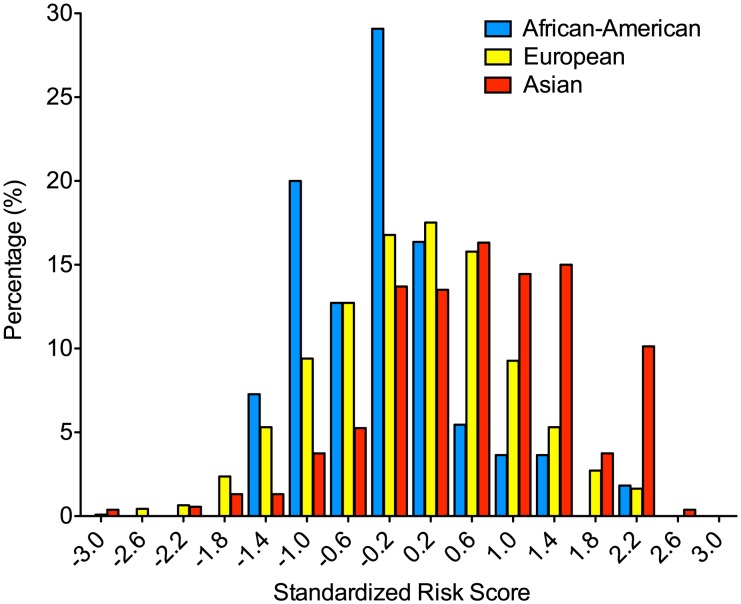
Differences in the distribution of the 7-SNP genetic risk score by ethnicity. Only healthy control participants of the replication studies that were fully genotyped at all 7 loci were used in this analysis. Similar to the GWAS study, the risk score distributions were significantly different by ethnicity (ANOVA p = 2.1×10^−38^). The corresponding differences in the distribution of risk alleles are depicted in Figure S1.

**Figure 3:**
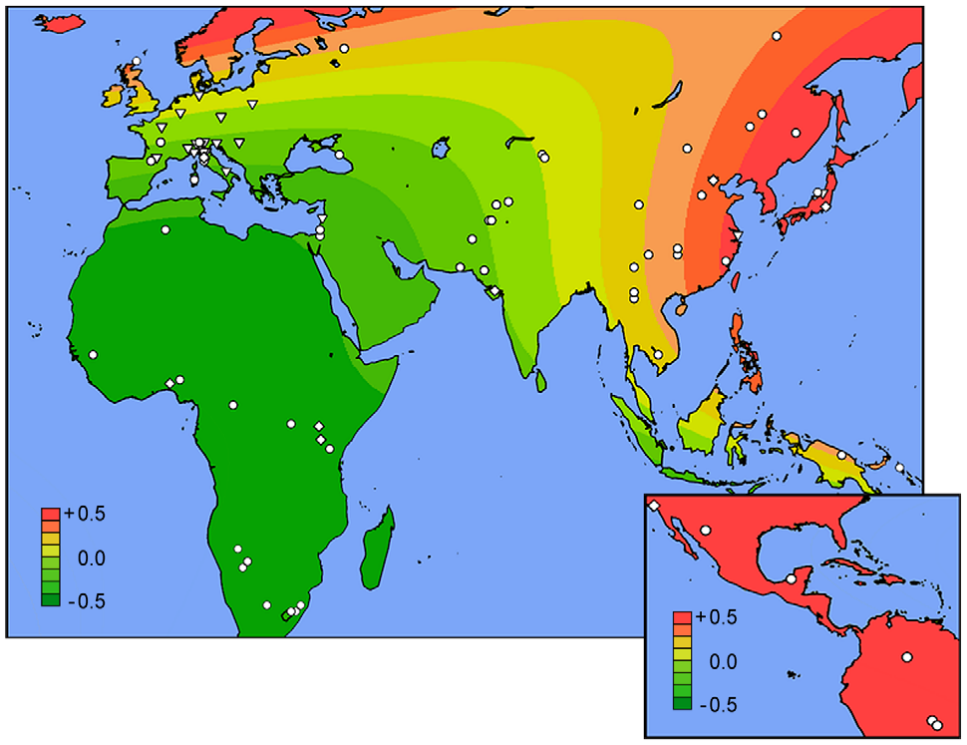
Worldwide geospatial risk analysis. Surface interpolation of the standardized risk score over Africa and Euroasia (main), and Americas (inset). Symbols represent the locations of sampled populations: HGDP (circles), HapMap-III (diamonds), and healthy controls from this study (triangles).

Similar to the GWAS study, we detected pronounced differences in the distributions of risk alleles among the three different ethnicities studied: for each of these seven risk loci, the frequency of the risk alleles was highest in East Asians and lowest in African-Americans (Figure S1). These differences were also reflected in highly significant disparities in the risk score distributions by ethnicity (Figure 2). Motivated by these observations, we examined global geographic variation in the genetic risk for IgAN by applying the newly refined IgAN risk score in 6,319 healthy individuals across 85 worldwide populations. We observed marked differences in the genetic risk across the world. Overall, the mean standardized risk score was lowest for Africans, intermediate for Middle Easterners and Europeans, and highest for East Asians and Native Americans (Figure 3 and Figure S2). Accordingly, the risk increased sharply with eastward distance from the prime meridian (Pearson's r = 0.27, p = 3.5×10^−108^). The same geospatial pattern were detected if we included only native populations of HGDP and HapMap-III (Figure S3), demonstrating that the findings are not biased by inclusion of control populations from the genetic association study. These data are consistent with the known East-West gradient in prevalence of IgAN, suggesting that genetic risk predicts prevalence.

**Figure 4:**
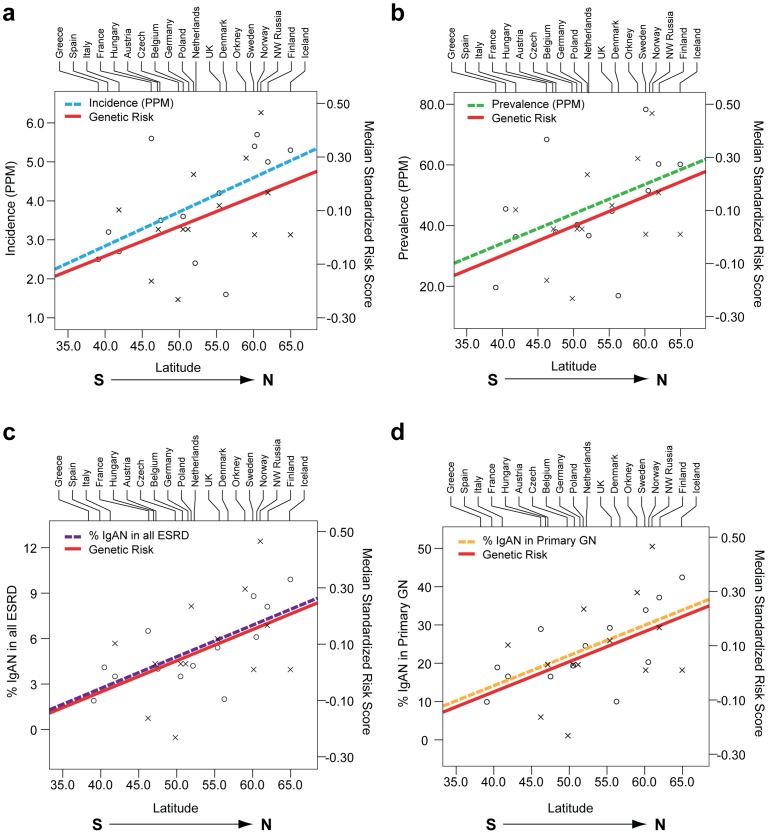
Correlation of average country latitude with country-specific genetic risk and IgAN–attributable ESRD across the European continent. The South to North latitude is indicated on the X-axis. The median genetic risk (x) is indicated on the right Y-axis. The following incidence and prevalence metrics (o) are indicated on the left Y-axis: (panel a) the incidence of ESRD due to IgAN per million population (correlation with latitude: r = 0.54, p = 0.05); (panel b) the prevalence of ESRD due to IgAN per million population (correlation with latitude: r = 0.47, p = 0.10); (panel c) the percent of IgAN patients among all ESRD cases (correlation with latitude: r = 0.67, p = 0.01); and (panel d) among ESRD cases due to primary glomerular disease (correlation with latitude: r = 0.71, p = 0.006). All p-values are derived based on a two-sided hypothesis test.

Unexpectedly, higher resolution analysis of the European continent revealed an additional increase in the risk from South to North (Pearson's r = 0.11, p = 1.3×10^−9^). For example, northwestern Russians and northern inhabitants of Orkney Islands (Scotland) have the highest risk scores when compared with the rest of the European continent (Tables S8 and S9). To confirm these finding and test whether North-South variation in genetic risk is also reflected in differences in IgAN occurrence, we obtained genetic data from additional European populations (Belgian, British, Finnish, Swedish and Icelandic) and compared genetic risk scores with the incidence and point prevalence of IgAN among end-stage renal disease (IgAN-ESRD) populations across Europe (Table S10). As predicted by the genetic risk score, our analysis confirmed a strong North-South cline of both incidence and prevalence across the European continent (Figure 4). Notably, this analysis includes only patients with end-stage IgAN, on dialysis or after kidney transplantation, thus it underestimates the true incidence and population prevalence of IgAN. Because the point prevalence of IgAN-ESRD (Figure 4b) can be confounded by differential survival on renal replacement therapy and differences in kidney biopsy practice by country, we also examined IgAN-ESRD prevalence expressed as a percentage of all ESRD (Figure 4c), and ESRD due to biopsy-diagnosed primary glomerulonephritis (Figure 4d). Regardless of the metric used to quantify differences in IgAN occurrence, regression of the genetic risk score and the prevalence data on the average latitude resulted in positive correlations and parallel trends.

**Figure 5:**
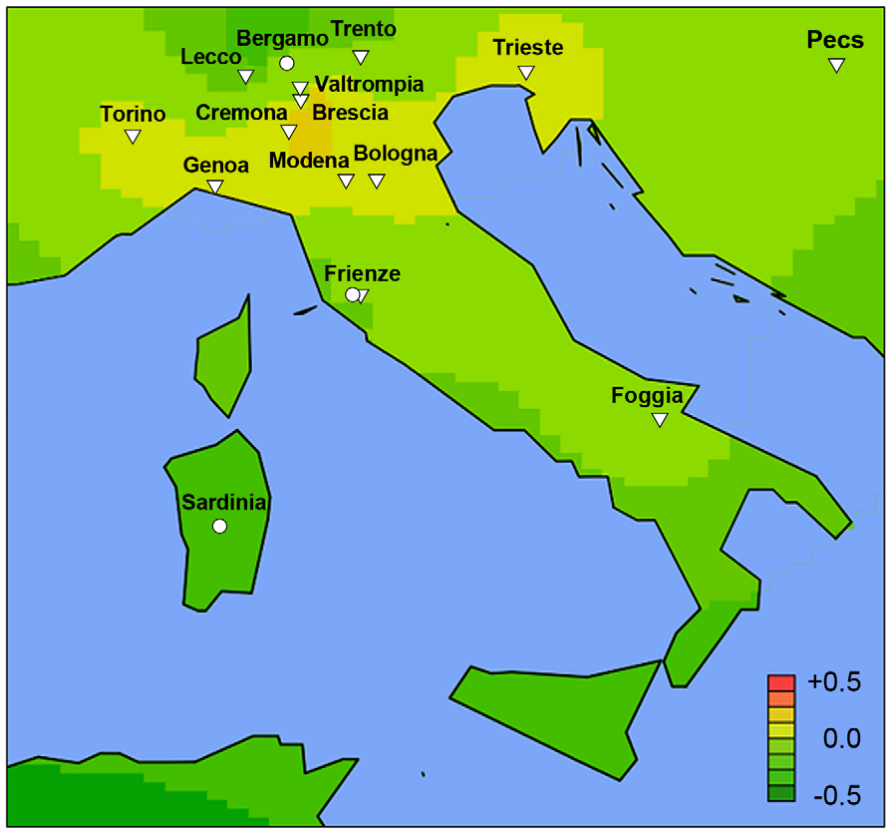
High-resolution geospatial risk analysis for Italy. A well defined region of higher genetic risk was uncovered in Northern Italy that centers on Valtrompia, Brescia, and Cremona (median standardized risk scores 0.31, 0.24 and 0.24, respectively). The healthy individuals from Valtrompia had the highest risk scores when compared to 16 other Italian populations sampled.

The co-variation in genetic risk score and IgAN-ESRD occurrence among world populations may also be in part influenced by differences in environment, or by other factors such as local medical guidelines for screening and treatment. To better distinguish these possibilities, we examined native populations that live under a uniform environment yet show variation in IgAN risk. In the densely sampled North Italian populations, the Alpine villagers of the Valtrompia region have a 3.5-fold higher prevalence of ESRD attributable to IgAN and primary glomerulonephritis when compared to the national average [16]. Consistent with this prevalence data, the median standardized risk score in this population was comparable to some of the Northern European countries and ranked as number one among the 17 Italian populations sampled in our study (Figure 5, Table S8).

**Figure 6:**
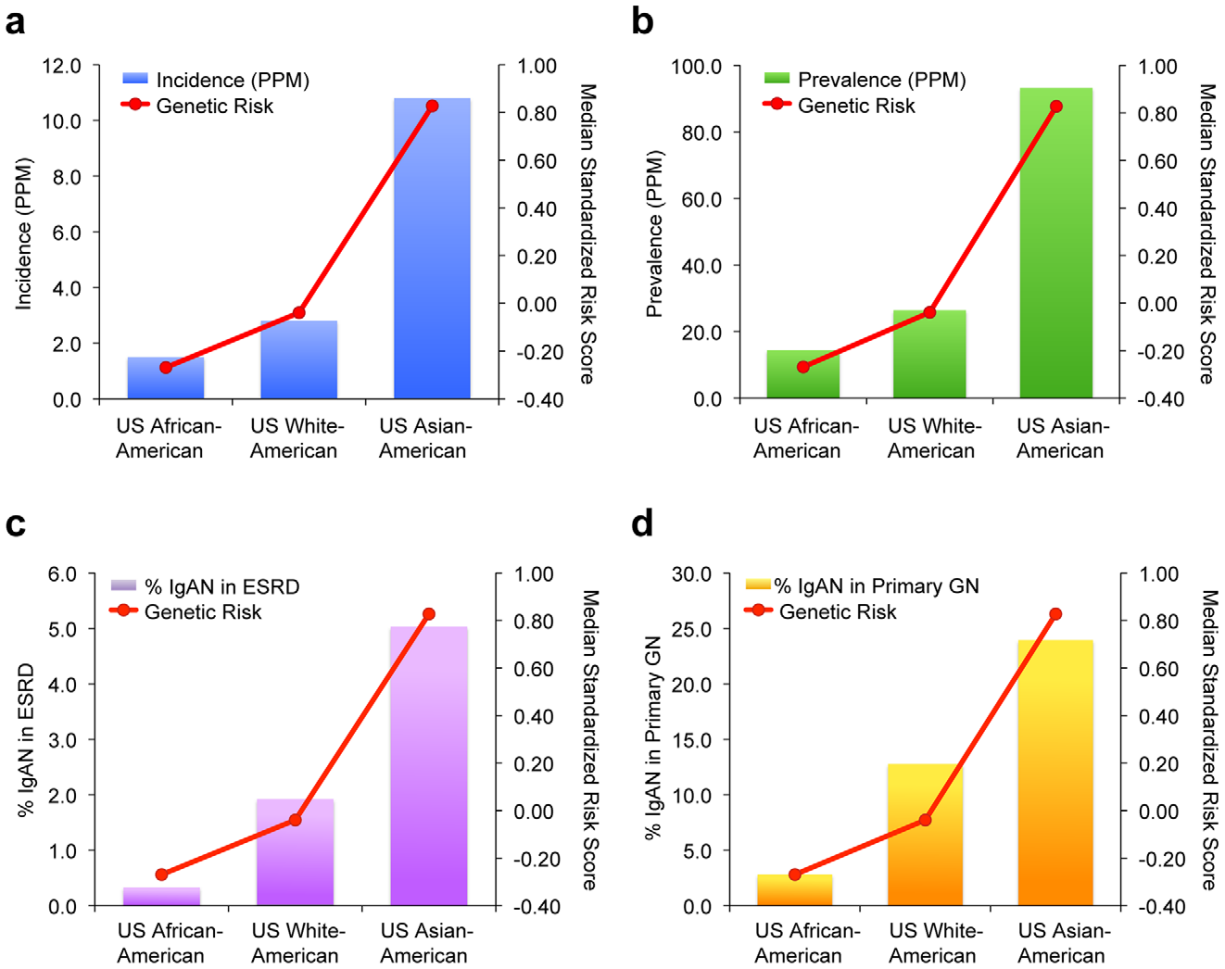
Genetic risk and IgAN–attributable ESRD among major US ethnicities. The relationship between IgAN risk scores (red line) and IgAN incidence and prevalence (bars) among US ethnicities are shown. The following metrics of IgAN occurrence are depicted: (panel a) the incidence of ESRD due to IgAN per million population by ethnicity, (panel b) the prevalence of ESRD due to IgAN per million population by ethnicity, (panel c) percent of IgAN among the total ESRD population by ethnicity; and (panel d) percent of IgAN among ESRD due to glomerular disease by ethnicity.

Conversely, we compared the genetic risk score and IgAN-ESRD prevalence in populations in the United States, where diverse ethnicities live under different environments and health care systems compared to the ancestral populations. The analysis of the USRDS dataset confirmed the striking ethnic differences in IgAN-ESRD prevalence (Table S11): the percentage of ESRD attributable to IgAN was 5-fold greater for Caucasian and 15-fold greater for Asian Americans compared to African-Americans. This increased IgAN-ESRD occurrence in Asian- compared to African-Americans far exceeds the 50% increase in risk predicted by genetic risk-score (one standard deviation difference), suggesting the presence of additional unaccounted genetic and environmental factors (Figure 6).

## Discussion

In this study, we examined the largest IgAN case-control cohorts reported to date. We first verified the five top signals identified in a recent GWAS for IgAN in independent cohorts and demonstrated robust replication of four loci, and heterogeneity at one locus. Using combined dataset of 10,755 individuals, we also identified novel risk alleles for IgAN in the *DQB1/DRB1* locus and detected a significant interaction between the *CFHR3/R1* and the *HORMAD2* loci. We also defined a more powerful genetic risk score that explained 4.7% in disease variance across all cohorts. Finally, in examination of 85 world populations, the genetic risk score paralleled the prevalence of IgAN, confirming the known East-West cline but also led to the detection of an association of IgAN-ESRD prevalence with latitude in Europe.

While ten of twelve tested SNPs (four susceptibility loci) were robustly replicated with direction-consistent ORs across all cohorts, the *TAP2/PSMB9* locus demonstrated moderately high level of heterogeneity. This locus remained genome-wide significant in the combined analyses under both fixed and random effects model. Family-based studies [17], [18], sperm typing experiments [19] and HapMap data have identified a recombination hotspot directly centered over the *TAP2* gene (22 cM/Mb, 5.5-kb centromeric from the 2 SNPs selected for replication). We can therefore hypothesize that high heterogeneity at this locus is due to the unusually high rates of recombination in this region, which perturbs LD patterns between tag-SNPs and causal variants; this situation has been shown to cause a “flip-flop” phenomenon in association results [20]. Therefore, higher density of SNP coverage on either side of the recombination hotspot will be needed to guide future replication and fine mapping efforts.

In addition to the independent replication of GWAS data, we identified two new signals in the *DQB1/DRB1* region that exhibit independent genome-wide significant effect in conditional analyses, providing support for multiple causal variants at this locus. These findings are consistent with previous studies of IgAN [15], [21] and other autoimmune diseases [22], [23], [24], [25], highlighting the complexity of associations in the MHC region. In our study, the strongest association signal originates in a protective haplotype tagged by rs9275596-C that carries HLA-DRB1*1501 and DQB1*602, also associated with protection against type I diabetes [24]. The causal variants underlying the other haplotypes remain obscure and their discovery will likely require comprehensive re-sequencing to define classical alleles.

Genetic interactions have been seldom described in association studies [26]. We detected a multiplicative interaction between the *CFHR3/R1* and the *HORMAD2* loci, which was most evident in the European cohorts, likely because the frequencies of both protective variants are considerably higher in this population. While this interaction was robust to multiple-testing correction for 7 SNPs, it will require confirmation in additional independent cohorts or via functional studies that examine whether these two loci are involved in a common biological pathway. Because the rs6677604-A allele tags a deletion in the *CFHR3/CFHR1* genes, this finding suggests that the absence of these proteins abrogates the benefit imparted by *HORMAD2* protective alleles. It is thus noteworthy that the *HORMAD2* locus encodes several cytokines (LIF, OSM) that can interact with complement factors [27].

A seven-SNP genetic risk score explained nearly 5% of IgAN variance and demonstrated co-variation with IgAN prevalence across multiple settings. The major limitations of geospatial modeling include variable sampling density and inadequate coverage of certain geographic regions. Using the most comprehensive resources presently available for geo-genetic analyses, we found that the genetic risk score strongly paralleled the well-known East-West gradient in IgAN prevalence [3], [28], [29], [30], [31], [32]. For each of these seven risk loci, the frequency of the risk alleles was highest in East Asians, lowest in African-Americans and intermediate in European populations. Accordingly, we detected co-variation of genetic risk with IgAN-ESRD incidence and prevalence among Asian-, White- and African-Americans, which share genetic background but not environment with their ancestral populations. Representative genetic data for U.S. Native Americans was not available from HGDP nor HapMap projects, precluding a direct comparison of their risk score with prevalence. However, the USRDS data and other reports indicate a high prevalence of IgAN-ESRD in US Native Americans [33], [34], [35], [36], [37], consistent with their ancestral origin from an Asian subpopulation that migrated across the Bering land bridge over 15,000 years ago [38]. In the more homogeneous population of Northern Italy, the median risk score in the Valtrompia valley was the highest among Northern Italian populations and comparable with the Northern European scores, consistent with Valtrompia's 3.5-fold higher prevalence of ESRD, which is largely attributable to IgAN [16]. Taken together, these data strongly suggested that variation in genetic risk partly explains the variation in geo-epidemiology of disease.

Because the genetic score captured general trends in IgAN epidemiology, we also tested whether the Northward gradient in genetic risk in Europe is mirrored by higher prevalence of kidney failure from IgAN. The ERA-EDTA data, which are the most unbiased source of information available, demonstrate that Nordic countries have over 2-fold higher incidence and prevalence of IgAN-ESRD compared to the Southern European countries. Although higher risk of IgAN in Northern Europe has not been previously appreciated, similar latitudinal risk gradients in prevalence and incidence have been well established for several other immune-mediated diseases, including type 1 diabetes [39], [40], multiple sclerosis [41], [42], and inflammatory bowel disease [43]. Interestingly, these disorders share risk alleles with IgAN, suggesting that variation in common genetic risk factors may mediate variation in prevalence of autoimmune disorders. Since our analysis was limited to prevalent IgAN-ESRD in countries with epidemiological data available and only a portion of IgAN cases progresses to ESRD, studies that can better estimate the population prevalence of all IgAN can confirm these findings and better delineate epidemiological connections to other immune mediated disorders.

The genetic and environmental factors leading to the observed geospatial pattern of genetic risk and disease prevalence are not clear. The pre-modern history of IgAN is not known because this disease was only first described in 1968 [44], shortly after the discovery and application of immunofluorescence in the analysis of kidney tissue. It is well known that mucosal infections can exacerbate disease, but specific environmental factors influencing the development of IgAN are not known. Based on a recently proposed pathogenesis model, the IgAN risk loci participate in sequential processes leading to the initiation and exacerbation of IgAN [45]. This may further explain the correlation of the genetic risk score with disease epidemiology. Interestingly, many of the IgAN loci are known to exhibit opposing effects on other autoimmune conditions [14]; for example, the *HLA-DQB1* and *HORMAD2* risk alleles are respectively protective for systemic lupus erythematosus, and inflammatory bowel disease. Thus balancing selection, in conjunction with local environmental factors, may be responsible for maintenance of risk alleles in different populations.

The current IgAN risk score captures a greater proportion of the disease variance compared to other GWAS for kidney functions, such as a recent study of 60,000 individual that reported 13 loci explaining only 1.4% of the variance for estimated glomerular filtration rate [46]. Nonetheless, the fraction of the IgAN variation explained remains modest. For example, the one standard deviation risk-score difference between Asian- and African-Americans predicts a 50% increase in risk, yet there is over 10-fold difference IgAN-ESRD occurrence between these two groups. These data suggest that additional genetic and environmental factors influence risk. Based on the effect sizes and allelic frequencies of the discovered SNPs, we estimate that doubling the GWAS sample size is likely to find up to 7 additional loci, while tripling the sample size would identify up to 11 additional loci at genome-wide significant p-values<10^−8^ (calculation performed as proposed by Park et al. [47]). Conditional analyses and higher-level interaction screens of more risk loci are likely to explain additional fraction of the missing heritability and better explain differences in population prevalence of this disease.

In summary, we report results of the largest collaborative genetic study of IgAN. We confirm that the IgAN risk loci discovered in GWAS explain a significant proportion of the disease risk worldwide and likely contribute to the geographic variation in disease prevalence. Our geospatial model suggests previously unrecognized northward risk gradient in Europe, which will require further confirmation by alternative sources of prevalence data, such as country specific biopsy-registry data or kidney donor-biopsy series. The approach presented in this study may serve as a blueprint for geo-genetic modeling of other complex traits that exhibit marked geographic differences in prevalence.

## Materials and Methods

### Ethics Statement

This investigation was conducted according to the principles expressed in the Declaration of Helsinki. All subjects provided informed consent to participate in genetic studies and the Institutional Review Board of Columbia University as well as local ethic review committees for each of the individual cohorts approved our study protocol.

### Study Cohorts

The case-control cohorts analyzed in this study were contributed by clinical nephrology centers across Europe, Asia, and North America (Table S1). All cases carried a biopsy diagnosis of IgAN defined by typical light microscopy features and predominant IgA staining on kidney tissue immunofluorescence, in the absence of liver disease or other autoimmune conditions. Each individual cohort of cases was accompanied by a control cohort of similar size, matched based on self-reported ethnicity and recruited from the same clinical center. The French cohort was composed of two sub-cohorts: the St. Etienne cohort recruited in the University North Hospital of Saint Etienne (289 cases and 244 controls), and the GN-Progress cohort recruited from the nephrology departments of the Paris region (207 cases and 159 controls). The Italian cohort was also composed of two sub-cohorts: the North Italian cohort recruited in the clinical centers of Genova, Torino, Brescia, Trento, Modena, Bologna, and Trieste (410 cases and 524 controls), and the South Italian cohort recruited in Foggia (81 cases and 80 controls). The German cohorts also represent two recruitment sites: the Stop-IgAN cohort recruited among the participants of the Stop-IgAN clinical trial based in Aachen (150 cases and 293 controls), and the Hamburg-Eppendorf cohort from northern Germany (101 cases and 80 controls). The Czech and the Hungarian cohorts were recruited through the Department of Nephrology, 1st Faculty of Medicine and General University Hospital, Charles University in Prague (245 cases and 223 controls) and the Nephrology Department of the University of Pécs (139 cases and 305 controls), respectively. The Japanese participants (264 cases and 294 controls) were recruited by the nephrologists of Niigata University. The Beijing cohort (333 cases and 289 controls) was recruited by the Renal Division of the Peking University First Hospital. Finally, our African-American cohort (34 cases and 60 controls) was recruited at Columbia University (New York, NY) and at the University of Alabama (Birmingham, AL). This smaller cohort is unique, as IgAN is exceedingly rare among individuals of African ancestry. In total, 2,253 cases and 2,621 controls were available for genotyping in the replication study. The composition and recruitment of the GWAS cohorts have been discussed in detail elsewhere [14].

### Genotyping and Genotype Quality Control

The genotyping was performed by KBiosciences (Hoddeston, England). and genotype calls were determined using an automated clustering algorithm the (SNP Viewer v.1.99, KBiosciences, 2008). The genotype clusters were also examined visually across all plates, to assure lack of technical artifacts. The overall genotyping rate across all samples was 98.2%. For quality control we calculated minor allele frequencies, as well as per-SNP and per-individual rates of missingness within each case-control cohort separately. Additionally, we tested for Hardy-Weinberg equilibrium among the control groups from each cohort to assure lack of bias due to genotyping artifacts or population stratification. All SNPs included in the final analyses had minor allele frequency greater than 1%, per-SNP missingness rate less than 5%, and all passed the HWE test in controls (p>1×10^−2^). Individuals with more than 2 missing genotypes out of the 12 loci were also excluded from the analysis.

The participants of the smaller GN-Progress study (207 cases and 159 controls) were genotyped using the Illumina HumanCNV370-duo chip at the Centre National de Génotypage (CEA, Evry, France). The analysis of intensity clusters and genotype calls were performed using the Illumina Genome Studio software. Of 366 genotyped individuals, two cases and 1.8% of SNPs were excluded based on low call rates (<95%). The overall genotyping rate was 99.6%. In total, 6 of 12 SNPs analyzed for replication were also present on the Illumina HumanCNV370-duo chip. The genotypes at the reminder loci were imputed using the phased HapMap-III CEU reference dataset (see Web Resources). The imputation was performed simultaneously for cases and controls, using MACH 1.0 software (see Web Resources). We used a standard single-step imputation approach, with 60 rounds of Markov Chain iterations to estimate the crossover maps, error rate maps, and all missing genotypes across each analyzed locus. The imputed SNPs had an excellent imputation quality, with an average estimated correlation between imputed genotypes and experimental genotypes of 0.98 (range 0.94–1.0). Consequently, association analyses using either the allelic dosage approach that accounts for imputation uncertainty, or the most likely genotype approach yielded similar results. Therefore, the most probable genotype calls were used in the downstream analyses. In the final quality control step, we compared the allelic frequencies and effect estimates between the two French cohorts (GN-Progress and St. Etienne). For each locus, we observed nearly identical frequencies among cases and controls and the odds ratios were homogenous between the two cohorts. The formal heterogeneity tests were not statistically significant for any of the tested loci (Cochrane's Q-test P>0.05, average I^2^ = 0). Therefore, these two cohorts were combined into a single cohort of 493 cases and 402 controls.

Similarly to the French cohorts, there was no significant heterogeneity at any of the loci for the two smaller German cohorts (STOP-IgAN and Hamburg-Eppendorf), and these were also combined into a single cohort of 249 cases and 372 controls. Analysis of the Northern and Southern Italian cohorts suggested some heterogeneity at 3 out of 12 SNPs (I^2^ = 40–50%). Although these observations were not statistically significant (Q-test P>0.05), we used a conservative stratified approach for all downstream analyses for these two cohorts. The final summary of all study cohorts before and after quality control is provided in Table S1.

### Power Calculation

We performed a power calculation for the final replication cohort size of 4,789 individuals (2,228 cases/2,561 controls) as a function of disease allele frequency and genotype relative risk (Table S2). The power was calculated in reference to a protective allele, with the range of allelic frequencies and effects comparable to the ones observed in the original GWAS. Assumptions included disease prevalence of 1%, log-additive model, no heterogeneity, and alpha = 0.01 (Bonferroni-adjusted considering five independent loci tested). This analysis confirmed that our study had ample power (nearly 100% for most loci) to replicate the associations observed in the initial GWAS. The power calculations were performed using QUANTO v.1.2 software [48].

### Association Analyses

The primary association analyses were performed using PLINK version 1.07 [49]. Similar to GWAS, we selected a standard 1-df Cochran-Armitage trend test as the primary association test. We also estimated the per-allele odds ratios and 95% confidence intervals for all tested SNPs within each individual cohort. The results across multiple cohorts were combined using an inverse variance-weighted method under a fixed-effects model (PLINK), as well as using a random effects model as proposed by Han and Eskin (METASOFT) [50]. We also tested for heterogeneity across cohorts by performing a formal Cochrane's Q heterogeneity test as well as by estimating the heterogeneity index (I^2^) [51].

### Conditional Analyses

The conditional association tests of the HLA loci were performed after controlling for the genotypes of the conditioning SNPs within each cohort using logistic regression (PLINK). The adjusted (conditioned) effect estimates were then combined across cohorts using a fixed effect meta-analysis considering no significant heterogeneity across these loci. For the purpose of validation of this approach, we also combined the results by adding cohort information as an additional covariate in the stratified analysis within the logistic regression framework. As expected, the results of both approaches were similar.

### Haplotype-Based Association Tests

These analyses were carried out in PLINK v1.07 [49]. Haplotypes were first phased using EM algorithm across the *HLA-DQB1, HLA-DQA1, HLA-DRB1* region. The haplotype frequencies were estimated in the cases and controls separately, as well as jointly in the entire cohort. Only common haplotypes with overall frequency >1% were included in the association tests. Global haplotype association test was performed using a *χ* ^2^ test with n-1 degrees of freedom for n common haplotype groups. The ORs and the corresponding 95% confidence intervals were estimated in reference to the most common haplotype (GCAT, frequency ∼35%).

### First-Order Interaction Analyses

To explore the possibility of interactions between the 7 independent risk variants, we screened all possible pairwise interaction terms for association with disease within the framework of logistic regression models (R version 2.10). As a screening test, we used 1-df LRT to compare two nested models: one with main effects only and one with main effects and a multiplicative (logit-additive) interaction term. We included cohort membership as a fixed covariate in both of these models. For this analysis we selected a Bonferroni-adjusted significance of 2.4×10^−3^, a conservative threshold that accounts for all 21 pairwise interaction terms tested. Significant interactions from this analysis were also tested using a 4-df genotypic interaction test. In this test, we compared a model with allelic effects, dominant effects, and their interaction terms with a reduced model with no interaction terms. We followed the coding proposed by Cordell and Clayton: for each SNP *i* we modeled its allelic effect x*_ia_* by coding the genotypes AA, AB, and BB as x*_ia_* = −1, 0, 1; we modeled dominance effects as x*_id_* = −0.5, 0.5, −0.5 for the genotypes AA, AB, and BB, respectively [52].

### Distributions of Protective Alleles and Risk Score Analyses

Each study participant was scored for the number of risk alleles and the distributions of protective alleles were compared between cohorts of different ethnicity. Only individuals with complete genotype information at the 7 scored loci (14 alleles) were included in this analysis. The distributions were analyzed separately for cases and controls. A *χ* ^2^ goodness-of-fit test was used to derive p-values for comparison of distributions. Because of a relatively small number of individuals at the tails of the distributions, for the purpose of statistical testing the tails of the distributions were binned into single-bin categories to achieve expected cell counts >5.

To confirm the results of conditional analyses and refine the genetic risk score proposed in the original GWAS, we subjected the genotype data from the entire cohort to a stepwise regression algorithm that selects significant covariates for the best predictive regression model based on Bayesian Information Criterion (the *step* function, R version 2.10). At model entry, we included all 12 genotyped SNPs, all 21 tested interactions, as well as cohort membership as a fixed covariate. Consistent with the results of our conditional analysis, the stepwise algorithm retained only the 7 SNPs exhibiting an independent effect along with the rs6677604*rs2412971 interaction term. All other terms were automatically dropped from the regression model.

The risk score was calculated as a weighted sum of the number of protective alleles at each locus multiplied by the log of the OR for each of the individual loci from the final fully adjusted model. Only individuals with non-missing genotypes for all 14 alleles were included in this analysis. The risk score was standardized across all populations using a z-score transformation, thus the standardized score represented the distance between the raw score and the population mean in units of standard deviation. The percentage of the total variance in disease state explained by the risk score was estimated by Nagelkerke's pseudo *R* ^2^ from the logistic regression model with the risk score as a quantitative predictor and disease state as an outcome. The C-statistic was estimated as an area under the receiver operating characteristic curve provided by the above logistic model. These analyses were carried out with SPSS Statistics version 19.0.

### Geospatial Analyses

For this purpose, we used publicly available genotype data of the Human Genome Diversity Panel (HGDP; 1,050 individuals representative of 52 worldwide populations), HapMap III (1,184 individuals representative of 11 populations), along with healthy controls genotyped as part of this study (4,547 individuals representative of 25 recruitment sites). The HGDP individuals have been previously genotyped for 660,918 markers using Illumina 650Y arrays (Stanford University). First, SNPs with genotyping rate<95% and samples with an overall call rate<98.5% were removed from the genome-wide data. Only 1,042 individuals with all 14 non-missing alleles at the 7 analyzed risk score loci were included in the final analysis. The geographic coordinates for the HGDP populations were downloaded from the CEPH website (see Web Resources). The HapMap III genotype data have been generated using two platforms: the Illumina Human1M (Wellcome Trust Sanger Institute) and the Affymetrix SNP 6.0 (Broad Institute). These files were merged into a single dataset of 1,440,616 markers, from which we removed (1) SNPs with genotyping rate<95%, (2) samples with an overall call rate<98.5%, (3) all non-founders from mother-father-child trios, and (4) individuals with missing genotypes at any of the 7 SNP loci used for risk scoring. In the global geospatial analyses, we excluded US-recruited individuals of African American (ASW), European (CEU), and Asian (CHD) ancestry considering non-specific geographic origin of these populations. However, the population of Guajarti Indians recruited in Houston (GIH) was mapped to the northwestern part of the Indian subcontinent, as these individuals reported having at least three out of four Gujarati grandparents, speak the Gujarati language, and trace their ancestry to the region of Gujarat. In total, 730 HapMap III individuals representative of 8 populations met our selection criteria and were included in the final analysis.

Because many European populations are underrepresented in HGDP and HapMap III datasets, we also included a total of 4,462 healthy controls from the GWAS and replication studies that were collected across 25 recruitment centers participating in our studies. Similar to the above criteria, only individuals with non-missing genotypes at all 7 scored SNPs were included in this analysis. The geographic coordinates for our populations were based on the location of recruitment centers and determined with Google Earth (see Web Resources). This resulted in a final dataset of 6,319 individuals sampled across 85 worldwide populations for geospatial analysis.

We fitted a 3^rd^ degree polynomial trend surface based on the latitude, longitude, and median standardized risk score for each of the 85 populations using least squares approach (Spatial package version 7.3-2, R version 2.10). For higher resolution maps, we used kriging technique and accounted for the possibility of spatial correlation of errors among more densely sampled populations by modeling the covariance function in an exponential form. The estimated risk surfaces were projected over the major continents using Maps package version 2.1–6 (R version 2.10).

### Analysis of Prevalence and Incidence Data

We obtained case counts of prevalent and incident ESRD stratified by primary renal diagnosis and by ethnicity from the United States Renal Data Systems (2011 USRDS Data Atlas, see Web Resources). For Europe, we obtained prevalent and incident ESRD case counts from the European Renal Association and European Dialysis and Transplant Association (ERA-EDTA Renal Registry, see Web Resources). Comprehensive data were available for a total of 13 European countries participating in this registry. We calculated the prevalence of ESRD due to IgAN using three definitions: (1) proportion of all ESRD cases attributable to IgAN, (2) proportion of all ESRD cases from primary glomerulonephritis attributable to IgAN, and (3) total number of ESRD cases due to IgAN per million population (PMP). The prevalence data for both USRDS and ERA-EDTA datasets were calculated for the same timepoint of December 31^st^, 2009. The incidence of ESRD due to IgAN was estimated using all the available data over a 3-year period for the ERA-EDTA registry (2007–2009), and a 5-year period for the USRDS registry (2005–2009). For correlation of genetic risk score with disease prevalence in the US, we scored representative samples of the three major US ethnic groups: 303 US Caucasians (CEU founders from HapMap-3 and healthy US controls from our original GWAS), 103 African-Americans (ASW founders from HapMap-3 and healthy controls from this study), and 74 Asian-Americans (CHD founders from HapMap-3). For correlation of genetic risk with disease prevalence in Europe, we calculated median standardized risk scores at a country level for 13 European countries for which we obtained genotype data. We confirmed the South-North disease gradient by regressing the prevalence and risk score data against each country's average latitude. The correlation and regression analyses were conducted in SPSS Statistics version 19.0.

### Web Resources

HAPMAP PHASE III Data: http://hapmap.ncbi.nlm.nih.gov/downloads/phasing/2009-02_phaseIII

HGDP Genotype Data: http://hagsc.org/hgdp

HGDP Population Data: http://www.cephb.fr/en/hgdp

MACH: http://www.sph.umich.edu/csg/abecasis/MaCH

PLINK: http://pngu.mgh.harvard.edu/~purcell/plink

METASOFT: http://genetics.cs.ucla.edu/meta

CRAN: http://cran.r-project.org

GOOGLE EARTH: http://www.google.com/earth

SPATIAL: http://cran.r-project.org/web/packages/spatial

MAPS: http://cran.r-project.org/web/packages/maps

USRDS Data Atlas 2011: http://www.usrds.org/atlas.aspx

ERA-EDTA Registry Annual Report 2009: http://www.era-edta-reg.org

## Supporting Information

Figure S1 Differences in the distributions of risk alleles at the 7 susceptibility loci among major ethnicities in the replication cohorts. Similar to the GWAS study, the distribution of the risk alleles differed by ethnicity: Asian controls carry more risk alleles compared to healthy Europeans or African-Americans (p = 3×10^−55^ and p = 5×10^−7^, respectively); European controls have more risk alleles compared to African-Americans (p = 6×10^−3^).
(PDF)

Figure S2 Inter-continental differences in the genetic risk score based on 85 worldwide populations used for geospatial analysis.
(PDF)

Figure S3 Geospatial risk model for native populations. Surface interpolation of the standardized risk score for HGDP (circles) and HapMap-III (diamonds) datasets. The risk increases globally with the distance from the prime meridian (Pearson's r = 0.31, p<2.2×10^−16^) and northward within Europe (Pearson's r = 0.13, p = 6.6×10^−4^).
(PDF)

Table S1 Summary of the case-control replication cohorts before and after quality control measures.
(PDF)

Table S2 Power calculation. Study power for the replication cohort of 4,789 individuals (2,228 cases/2,561 controls) as a function of disease allele frequency and genotype relative risk. The power was calculated in reference to a protective allele; the range of allelic frequencies and effects was based on the results of the original GWAS. Assumptions include: disease prevalence of 1%, log-additive model, no heterogeneity, and alpha = 0.01 (Bonferroni-adjusted considering five independent loci tested).
(PDF)

Table S3 Case-control association results for the individual replication cohorts.
(PDF)

Table S4 Pairwise LD between the SNPs of the HLA region: r2 (top right half) and D′ (bottom left half) for all cohorts (top), Europeans (middle) and Asians (bottom).
(PDF)

Table S5 Haplotype analysis of rs9275224, rs2856717, rs9275424, and rs9275596 at the *HLA- DQB1/DRB1* locus. The most common haplotype of 4 major alleles (GCAT) is used as a reference to derive odds ratios for all other haplotypes. Only common haplotypes (frequency>1%) are tested for association.
(PDF)

Table S6 All possible 1^st^ order multiplicative interactions between the 7 SNPs with independent effects on disease risk. Statistical significance is assessed using a Bonferroni-corrected threshold, alpha 0.05/21 = 2.4×10^−3^.
(PDF)

Table S7 The comparison of the original and the newly refined genetic risk score.
(PDF)

Table S8 African, Middle Eastern, and European populations included in the geospatial risk analysis. The populations were grouped by their continental origin and sorted based on the median genetic risk score.
(PDF)

Table S9 Asian, Oceanian, and American populations included in the geospatial risk analysis. The populations were grouped by their continental origin and sorted based on the median genetic risk score.
(PDF)

Table S10 Prevalence and Incidence of ESRD due to IgAN in Europe. Primary data obtained from the ERA-EDTA Registry.
(PDF)

Table S11 Prevalence and Incidence of ESRD due to IgAN in the US. Primary data obtained from the USRDS Annual Report, 2011.
(PDF)
