# Supplementary material for: Geographic Differences in Genetic Susceptibility to IgA Nephropathy: GWAS Replication Study and Geospatial Risk Analysis
Source: PLoS Genet. 2012 Jun 21;8(6):e1002765. doi: 10.1371/journal.pgen.1002765 (PMC3380840; doi:10.1371/journal.pgen.1002765)

Supplemental Figure 1. Differences in the distributions of risk alleles at the 7 susceptibility loci among major ethnicities in the replication cohorts. Similar to the GWAS study, the distribution of the risk alleles differed by ethnicity: Asian controls carry more risk alleles compared to healthy Europeans or African-Americans ($p = 3 \times 10^{-55}$ and $p = 5 \times 10^{-7}$, respectively); European controls have more risk alleles compared to African-Americans ($p = 6 \times 10^{-3}$).

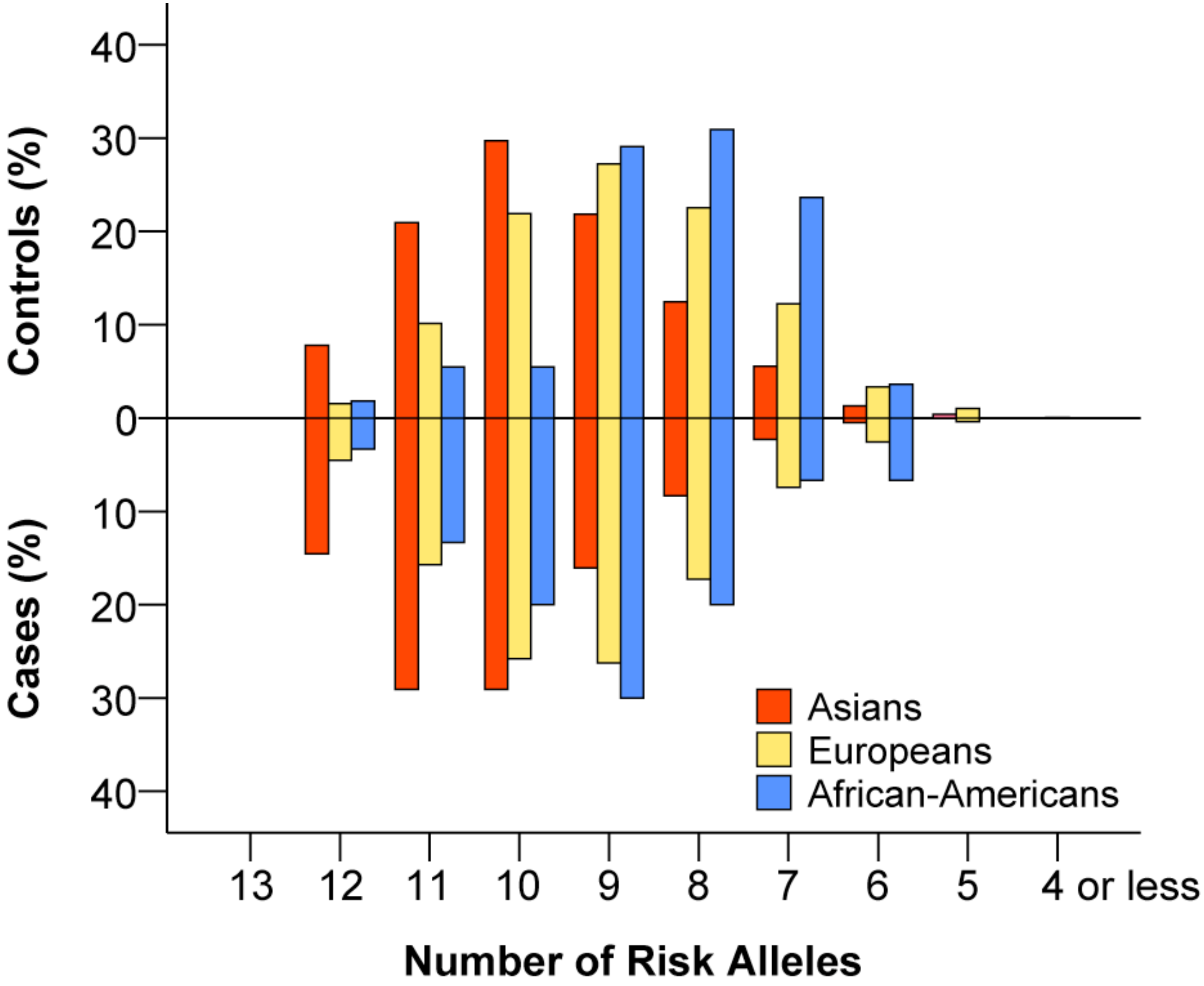

Supplement: Figure S1 — Differences in the distributions of risk alleles at the 7 susceptibility loci among major ethnicities in the replication cohorts. Similar to the GWAS study, the distribution of the risk alleles differed by ethnicity: Asian controls carry more risk alleles compared to healthy Europeans or African-Americans (p = 3×10^−55^ and p = 5×10^−7^, respectively); European controls have more risk alleles compared to African-Americans (p = 6×10^−3^). (PDF) [file pgen.1002765.s001.pdf]
