# Supplementary material for: Geographic Differences in Genetic Susceptibility to IgA Nephropathy: GWAS Replication Study and Geospatial Risk Analysis
Source: PLoS Genet. 2012 Jun 21;8(6):e1002765. doi: 10.1371/journal.pgen.1002765 (PMC3380840; doi:10.1371/journal.pgen.1002765)

Supplemental Figure 2. Inter-continental differences in the genetic risk score based on 85 worldwide populations used for geospatial analysis.

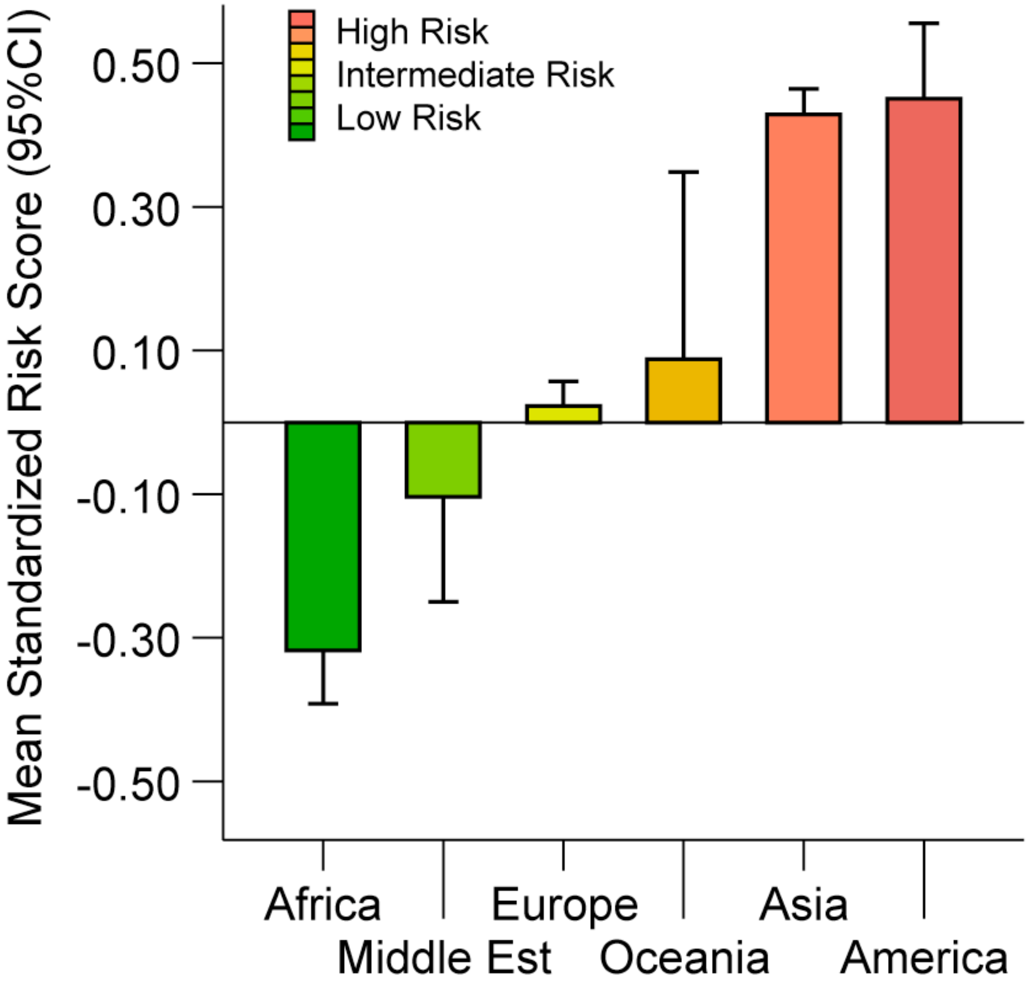

Supplement: Figure S2 — Inter-continental differences in the genetic risk score based on 85 worldwide populations used for geospatial analysis. (PDF) [file pgen.1002765.s002.pdf]
