# Supplementary material for: Geographic Differences in Genetic Susceptibility to IgA Nephropathy: GWAS Replication Study and Geospatial Risk Analysis
Source: PLoS Genet. 2012 Jun 21;8(6):e1002765. doi: 10.1371/journal.pgen.1002765 (PMC3380840; doi:10.1371/journal.pgen.1002765)

Supplemental Figure 3. Geospatial risk model for native populations. Surface interpolation of the standardized risk score for HGDP (circles) and HapMap-III (diamonds) datasets. The risk increases globally with the distance from the prime meridian (Pearson's $r = 0.31$, $p < 2.2 \times 10^{-16}$) and northward within Europe (Pearson's $r = 0.13$, $p = 6.6 \times 10^{-4}$).

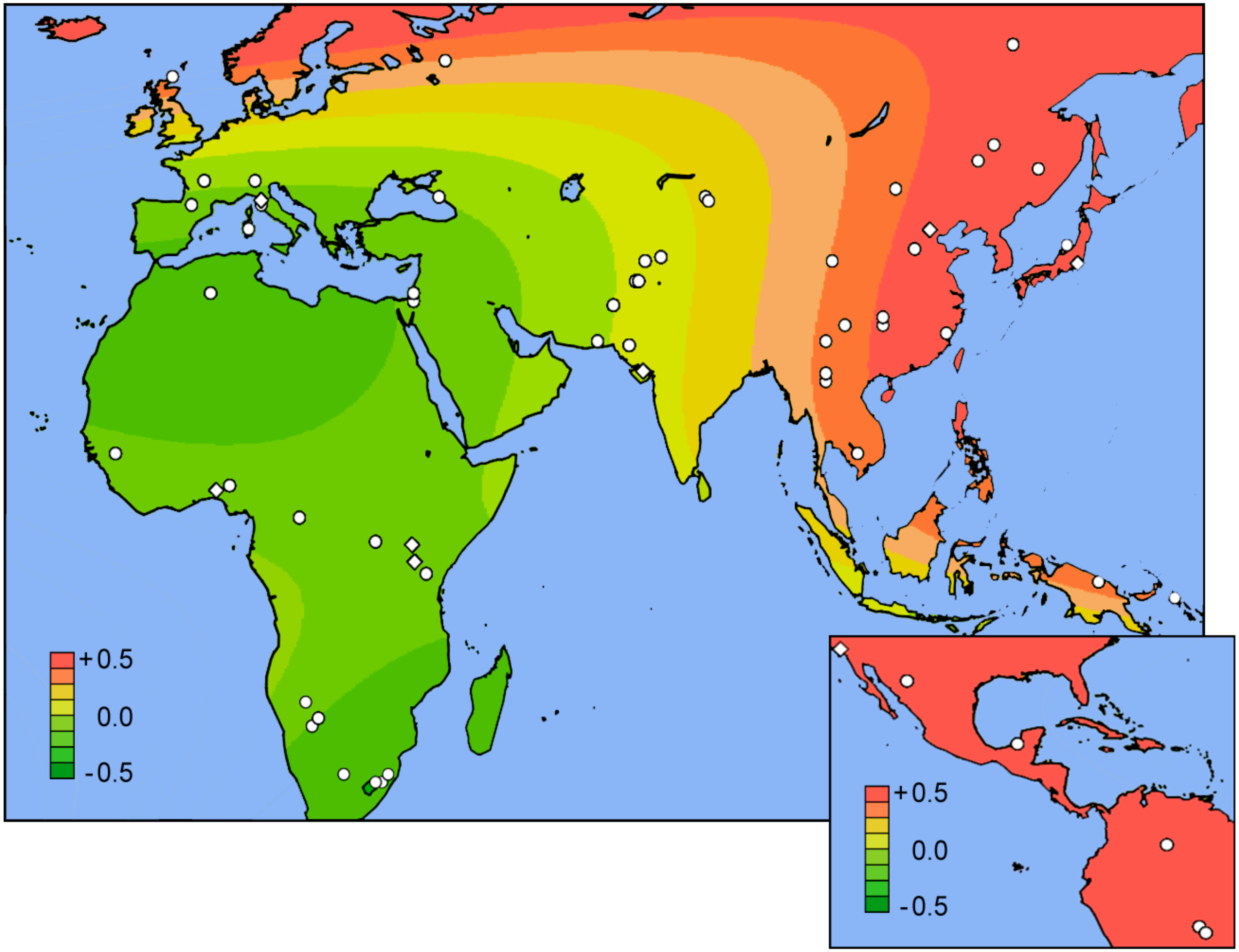

Supplement: Figure S3 — Geospatial risk model for native populations. Surface interpolation of the standardized risk score for HGDP (circles) and HapMap-III (diamonds) datasets. The risk increases globally with the distance from the prime meridian (Pearson's r = 0.31, p<2.2×10^−16^) and northward within Europe (Pearson's r = 0.13, p = 6.6×10^−4^). (PDF) [file pgen.1002765.s003.pdf]
