# Supplementary material for: Geographic Differences in Genetic Susceptibility to IgA Nephropathy: GWAS Replication Study and Geospatial Risk Analysis
Source: PLoS Genet. 2012 Jun 21;8(6):e1002765. doi: 10.1371/journal.pgen.1002765 (PMC3380840; doi:10.1371/journal.pgen.1002765)
Supplement: Table S1 — Summary of the case-control replication cohorts before and after quality control measures. (PDF) [file pgen.1002765.s004.pdf]

Supplemental Table 1. Summary of the case-control replication cohorts before and after quality control measures.

Cohorts by Nationality:	Genotyped		After QC		Average Genotype Call Rate[#]
	Total	Cases / Controls	Total	Cases / Controls	
Italian Cohort [*]	1,165	491 / 674	1,116	478 / 638	98.4%
French Cohort [**]	899	496 / 403	895	493 / 402	99.2%
German Cohort [***]	624	251 / 373	621	249 / 372	98.8%
Czech Cohort	468	245 / 223	465	244 / 221	97.8%
Hungarian Cohort	444	139 / 305	431	138 / 293	98.4%
Han Chinese Cohort	622	333 / 289	617	333 / 284	95.7%
Japanese Cohort	558	264 / 294	550	259 / 291	98.4%
African-American Cohort	94	34 / 60	94	34 / 60	98.9%
**All Cohorts Total:**	**4,874**	**2,253 / 2,621**	**4,789**	**2,228 / 2,561**	98.2%

* The Italian Cohort is composed of two sub-cohorts: the North Italian (410 cases and 524 controls) and the South Italian (81 cases and 150 controls).
** The French Cohort is composed of two sub-cohorts: the St.Etienne (289 cases and 244 controls) and the GN-Progress (207 cases and 159 controls).
*** The German Cohort is composed of two sub-cohorts: the Stop-IGAN (150 cases and 293 controls) and the Hamburg-Eppendorf (101 cases and 80 controls).
# For combined datasets, the genotyping call rate represents a weighted average of the genotyping rates (weights based on cohort size).
