# Supplementary material for: Geographic Differences in Genetic Susceptibility to IgA Nephropathy: GWAS Replication Study and Geospatial Risk Analysis
Source: PLoS Genet. 2012 Jun 21;8(6):e1002765. doi: 10.1371/journal.pgen.1002765 (PMC3380840; doi:10.1371/journal.pgen.1002765)
Supplement: Table S2 — Power calculation. Study power for the replication cohort of 4,789 individuals (2,228 cases/2,561 controls) as a function of disease allele frequency and genotype relative risk. The power was calculated in reference to a protective allele; the range of allelic frequencies and effects was based on the results of the original GWAS. Assumptions include: disease prevalence of 1%, log-additive model, no heterogeneity, and alpha = 0.01 (Bonferroni-adjusted considering five independent loci tested). (PDF) [file pgen.1002765.s005.pdf]

Supplemental Table 2. Power calculation. Study power for the replication cohort of 4,789 individuals (2,228 cases / 2,561 controls) as a function of disease allele frequency and genotype relative risk. The power was calculated in reference to a protective allele; the range of allelic frequencies and effects was based on the results of the original GWAS. Assumptions include: disease prevalence of 1%, log-additive model, no heterogeneity, and alpha = 0.01 (Bonferroni-adjusted considering five independent loci tested).

	Relative Risk (Additive Model)			
**Disease Allele Frequency**	**0.50**	**0.60**	**0.70**	**0.80**
**0.10**	100%	100%	98.9%	70.7%
**0.20**	100%	100%	100%	94.8%
**0.30**	100%	100%	100%	98.9%
**0.40**	100%	100%	100%	99.6%
**0.50**	100%	100%	100%	99.8%
