# Supplementary material for: Geographic Differences in Genetic Susceptibility to IgA Nephropathy: GWAS Replication Study and Geospatial Risk Analysis
Source: PLoS Genet. 2012 Jun 21;8(6):e1002765. doi: 10.1371/journal.pgen.1002765 (PMC3380840; doi:10.1371/journal.pgen.1002765)
Supplement: Table S3 — Case-control association results for the individual replication cohorts. (PDF) [file pgen.1002765.s006.pdf]

Supplemental Table 3. Case-control association results for the individual replication cohorts.

			Italian Cohort N = 1,116 (478 cases / 638 controls)		French Cohort N = 895 (493 cases / 402 controls)		German Cohort N = 621 (249 cases / 372 controls)		Czech Cohort N = 465 (244 cases / 221 controls)		Hungarian Cohort N = 431 (138 cases / 293 controls)		Chinese Cohort N = 617 (333 cases / 284 controls)		Japanese Cohort N = 550 (259 cases / 291 controls)		African-American Cohort N = 94 (34 cases / 60 controls)	
**Chr**	**Loc (kb)**	**SNP (minor allele)**	**OR**	**P-value**	**OR**	**P-value**	**OR**	**P-value**	**OR**	**P-value**	**OR**	**P-value**	**OR**	**P-value**	**OR**	**P-value**	**OR**	**P-value**
1	194,918	**rs3766404 (C)**	0.85	2.0×10^{-1}	0.72	6.4×10^{-2}	0.71	3.8×10^{-2}	1.01	9.5×10^{-1}	0.79	2.7×10^{-1}	0.68	7.7×10^{-2}	0.72	1.8×10^{-1}	0.60	1.2×10^{-1}
1	194,953	**rs6677604 (A)**	0.88	2.2×10^{-1}	0.70	4.3×10^{-3}	0.74	3.5×10^{-2}	1.02	9.2×10^{-1}	0.73	9.4×10^{-2}	0.66	8.6×10^{-2}	0.71	2.6×10^{-1}	0.59	1.1×10^{-1}
6	32,768	**rs9275224 (A)**	0.78	6.4×10^{-3}	0.76	4.7×10^{-3}	0.71	4.4×10^{-3}	0.84	2.3×10^{-1}	0.60	9.5×10^{-4}	0.80	6.2×10^{-2}	0.74	1.8×10^{-2}	0.60	1.1×10^{-1}
6	32,778	**rs2856717 (T)**	0.91	3.5×10^{-1}	0.89	2.7×10^{-1}	0.87	2.6×10^{-1}	0.89	3.6×10^{-1}	0.65	6.7×10^{-3}	0.94	6.5×10^{-1}	0.76	9.7×10^{-2}	0.71	3.5×10^{-1}
6	32,779	**rs9275424 (G)**	0.99	9.6×10^{-1}	1.36	6.0×10^{-3}	1.20	1.8×10^{-1}	1.21	2.5×10^{-1}	1.37	9.6×10^{-2}	1.14	4.1×10^{-1}	1.47	2.6×10^{-3}	0.82	5.6×10^{-1}
6	32,789	**rs9275596 (C)**	0.78	2.1×10^{-2}	0.82	6.5×10^{-2}	0.82	1.2×10^{-1}	0.73	6.7×10^{-2}	0.58	1.2×10^{-3}	0.70	1.5×10^{-2}	0.66	3.9×10^{-2}	0.78	4.8×10^{-1}
6	32,917	**rs9357155 (A)**	0.76	5.9×10^{-2}	1.23	1.6×10^{-1}	0.71	6.0×10^{-2}	0.79	2.4×10^{-1}	1.59	5.4×10^{-2}	1.23	1.9×10^{-1}	0.83	3.2×10^{-1}	1.56	4.2×10^{-1}
6	32,919	**rs2071543 (A)**	0.82	1.6×10^{-1}	1.11	4.7×10^{-1}	0.67	2.8×10^{-2}	0.71	1.1×10^{-1}	1.58	5.3×10^{-2}	1.17	5.1×10^{-1}	0.80	2.1×10^{-1}	1.26	6.0×10^{-1}
6	33,194	**rs1883414 (T)**	0.90	2.6×10^{-1}	0.84	8.1×10^{-2}	1.00	9.9×10^{-1}	0.80	1.2×10^{-1}	0.90	5.1×10^{-1}	0.83	1.8×10^{-1}	0.84	2.5×10^{-1}	0.70	3.9×10^{-1}
6	33,205	**rs3129269 (T)**	0.94	4.9×10^{-1}	0.92	4.1×10^{-1}	1.01	9.5×10^{-1}	0.76	5.7×10^{-2}	0.96	7.9×10^{-1}	0.82	1.5×10^{-1}	0.74	7.2×10^{-2}	0.74	4.4×10^{-1}
22	28,824	**rs2412971 (A)**	0.85	6.7×10^{-2}	0.81	2.2×10^{-2}	0.86	1.8×10^{-1}	0.92	5.1×10^{-1}	0.97	8.4×10^{-1}	0.72	9.5×10^{-3}	0.57	1.7×10^{-4}	0.62	1.3×10^{-1}
22	28,859	**rs2412973 (A)**	0.85	5.8×10^{-2}	0.79	1.4×10^{-2}	0.86	1.7×10^{-1}	0.93	5.5×10^{-1}	0.98	8.9×10^{-1}	0.72	9.8×10^{-3}	0.57	1.3×10^{-4}	0.62	1.3×10^{-1}
