# Supplementary material for: Geographic Differences in Genetic Susceptibility to IgA Nephropathy: GWAS Replication Study and Geospatial Risk Analysis
Source: PLoS Genet. 2012 Jun 21;8(6):e1002765. doi: 10.1371/journal.pgen.1002765 (PMC3380840; doi:10.1371/journal.pgen.1002765)
Supplement: Table S4 — Pairwise LD between the SNPs of the HLA region: r2 (top right half) and D′ (bottom left half) for all cohorts (top), Europeans (middle) and Asians (bottom). (PDF) [file pgen.1002765.s007.pdf]

Supplemental Table 4. Pairwise LD between the SNPs of the HLA region: r2 (top right half) and D' (bottom left half) for all cohorts (top), Europeans (middle) and Asians (bottom).

All cohorts: N = 10,755

r^2 / D'	rs9275224	rs2856717	rs9275424	rs9275596	rs9357155	rs1883414
**rs9275224**		0.543	0.212	0.387	0.000	0.014
**rs2856717**	0.998		0.117	0.715	0.004	0.001
**rs9275424**	0.997	0.997		0.091	0.000	0.003
**rs9275596**	0.946	0.947	0.993		0.002	0.001
**rs9357155**	0.049	0.101	0.013	0.066		0.001
**rs1883414**	0.159	0.030	0.163	0.039	0.108	

European Cohorts: N=5,938

r^2 / D'	rs9275224	rs2856717	rs9275424	rs9275596	rs9357155	rs1883414
**rs9275224**		0.623	0.205	0.451	0.005	0.000
**rs2856717**	0.998		0.130	0.715	0.000	0.002
**rs9275424**	0.997	0.998		0.104	0.007	0.000
**rs9275596**	0.943	0.938	0.994		0.002	0.002
**rs9357155**	0.229	0.041	0.127	0.206		0.003
**rs1883414**	0.028	0.093	0.029	0.115	0.101	

Asian Cohorts: N=4,723

r^2 / D'	rs9275224	rs2856717	rs9275424	rs9275596	rs9357155	rs1883414
**rs9275224**		0.437	0.217	0.300	0.003	0.050
**rs2856717**	0.997		0.096	0.699	0.037	0.004
**rs9275424**	0.998	1.000		0.072	0.010	0.008
**rs9275596**	0.950	0.959	0.995		0.039	0.008
**rs9357155**	0.102	0.224	0.381	0.200		0.007
**rs1883414**	0.343	0.065	0.286	0.105	0.381	
