# Supplementary material for: Geographic Differences in Genetic Susceptibility to IgA Nephropathy: GWAS Replication Study and Geospatial Risk Analysis
Source: PLoS Genet. 2012 Jun 21;8(6):e1002765. doi: 10.1371/journal.pgen.1002765 (PMC3380840; doi:10.1371/journal.pgen.1002765)
Supplement: Table S5 — Haplotype analysis of rs9275224, rs2856717, rs9275424, and rs9275596 at the HLA- DQB1/DRB1 locus. The most common haplotype of 4 major alleles (GCAT) is used as a reference to derive odds ratios for all other haplotypes. Only common haplotypes (frequency>1%) are tested for association. (PDF) [file pgen.1002765.s008.pdf]

Supplemental Table 5. Haplotype analysis of rs9275224, rs2856717, rs9275424, and rs9275596 at the *HLA- DQB1/DRB1* locus. The most common haplotype of 4 major alleles (GCAT) is used as a reference to derive odds ratios for all other haplotypes. Only common haplotypes (frequency >1%) are tested for association.

All Cohorts: N = 10,755

	Freq. Overall	Freq. Cases	Freq. Controls	OR	95%CI	P-global
**GCAT**	0.352	0.365	0.338	-reference-	-reference-	
**ATAC**	0.213	0.180	0.245	0.69	0.64 - 0.74	
**ACAT**	0.130	0.119	0.141	0.78	0.71 - 0.85	3×10^{-43}
**ATAT**	0.050	0.058	0.043	1.25	1.10 - 1.42	
**GCGT**	0.246	0.270	0.222	1.12	1.04 - 1.20	

European Cohorts: N = 5,938

	Freq. Overall	Freq. Cases	Freq. Controls	OR	95%CI	P-global
**GCAT**	0.350	0.365	0.336	-reference-	-reference-	
**ATAC**	0.256	0.228	0.282	0.75	0.68 - 0.82	
**ACAT**	0.104	0.090	0.117	0.71	0.62 - 0.81	3×10^{-18}
**ATAT**	0.054	0.062	0.047	1.21	1.02 - 1.43	
**GCGT**	0.224	0.244	0.205	1.09	0.99 - 1.20	

Asian Cohorts: N = 4,723

	Freq. Overall	Freq. Cases	Freq. Controls	OR	95%CI	P-global
**GCAT**	0.356	0.366	0.345	-reference-	-reference-	
**ATAC**	0.156	0.124	0.191	0.61	0.54 - 0.69	
**ACAT**	0.163	0.152	0.175	0.81	0.72 - 0.92	4×10^{-24}
**ATAT**	0.046	0.053	0.038	1.34	1.09 - 1.65	
**GCGT**	0.274	0.299	0.245	1.15	1.04 - 1.27	
