# Supplementary material for: Geographic Differences in Genetic Susceptibility to IgA Nephropathy: GWAS Replication Study and Geospatial Risk Analysis
Source: PLoS Genet. 2012 Jun 21;8(6):e1002765. doi: 10.1371/journal.pgen.1002765 (PMC3380840; doi:10.1371/journal.pgen.1002765)
Supplement: Table S6 — All possible 1st order multiplicative interactions between the 7 SNPs with independent effects on disease risk. Statistical significance is assessed using a Bonferroni-corrected threshold, alpha 0.05/21 = 2.4×10^−3^. (PDF) [file pgen.1002765.s009.pdf]

Supplemental Table 6. All possible 1st order multiplicative interactions between the 7 SNPs with independent effects on disease risk.

Statistical significance is assessed using a Bonferroni-corrected threshold, alpha 0.05/21=$2.4x10^{-3}$.

All cohorts: N = 10,755

p-value / beta	rs6677604 (A)	rs9275224 (A)	rs2856717 (T)	rs9275596 (C)	rs9357155 (A)	rs1883414 (T)	rs2412971 (A)
**rs6677604 (A)**		0.80 (NS)	0.08 (NS)	0.13 (NS)	0.62 (NS)	0.89 (NS)	**2.5×10^{-4} *****
**rs9275224 (A)**	0.01		0.26 (NS)	0.27 (NS)	0.52 (NS)	0.40 (NS)	0.27 (NS)
**rs2856717 (T)**	0.11	0.06		0.36 (NS)	0.90 (NS)	0.01 (NS)	0.02 (NS)
**rs9275596 (C)**	0.10	0.06	0.05		0.56 (NS)	0.01 (NS)	0.02 (NS)
**rs9357155 (A)**	0.04	0.04	0.01	-0.04		0.25 (NS)	0.09 (NS)
**rs1883414 (T)**	-0.01	0.04	0.13	0.14	0.07		0.01 (NS)
**rs2412971 (A)**	**0.21**	0.04	0.10	0.11	0.10	0.12	

European Cohorts: N=5,938

p-value / beta	rs6677604 (A)	rs9275224 (A)	rs2856717 (T)	rs9275596 (C)	rs9357155 (A)	rs1883414 (T)	rs2412971 (A)
**rs6677604 (A)**		0.36 (NS)	0.16 (NS)	0.48 (NS)	0.55 (NS)	0.30 (NS)	**1.4×10^{-3} *****
**rs9275224 (A)**	0.06		0.77 (NS)	0.96 (NS)	0.74 (NS)	0.37 (NS)	0.10 (NS)
**rs2856717 (T)**	0.10	0.02		0.98 (NS)	0.98 (NS)	0.07 (NS)	0.16 (NS)
**rs9275596 (C)**	0.05	0.00	0.00		0.59 (NS)	0.12 (NS)	0.39 (NS)
**rs9357155 (A)**	0.06	0.03	0.00	-0.05		0.19 (NS)	0.04 (NS)
**rs1883414 (T)**	-0.08	0.05	0.11	0.10	0.12		0.06 (NS)
**rs2412971 (A)**	**0.22**	0.09	0.08	0.05	0.17	0.11	

Asian Cohorts: N=4,723

p-value / beta	rs6677604 (A)	rs9275224 (A)	rs2856717 (T)	rs9275596 (C)	rs9357155 (A)	rs1883414 (T)	rs2412971 (A)
**rs6677604 (A)**		0.15 (NS)	0.71 (NS)	0.96 (NS)	0.23 (NS)	0.72 (NS)	**0.60 (NS)**
**rs9275224 (A)**	-0.20		0.34 (NS)	0.31 (NS)	0.91 (NS)	0.58 (NS)	0.47 (NS)
**rs2856717 (T)**	-0.06	0.09		0.75 (NS)	0.78 (NS)	0.24 (NS)	0.54 (NS)
**rs9275596 (C)**	-0.01	0.10	0.04		0.90 (NS)	0.07 (NS)	0.29 (NS)
**rs9357155 (A)**	-0.23	0.01	-0.03	-0.01		0.96 (NS)	0.61 (NS)
**rs1883414 (T)**	0.06	-0.04	0.10	0.16	0.01		0.31 (NS)
**rs2412971 (A)**	**0.07**	-0.05	0.05	0.09	-0.04	0.08	
