# Supplementary material for: Geographic Differences in Genetic Susceptibility to IgA Nephropathy: GWAS Replication Study and Geospatial Risk Analysis
Source: PLoS Genet. 2012 Jun 21;8(6):e1002765. doi: 10.1371/journal.pgen.1002765 (PMC3380840; doi:10.1371/journal.pgen.1002765)
Supplement: Table S7 — The comparison of the original and the newly refined genetic risk score. (PDF) [file pgen.1002765.s010.pdf]

Supplemental Table 7. The comparison of the original and the newly refined genetic risk score.

		Original Risk Score				Newly Refined Risk Score			
**Cohort**	**N#**	**R^{2*}**	**C (95%CI)****	**OR (95%CI)*****	**P-value******	**R^{2*}**	**C (95%CI)****	**OR (95%CI)*****	**P-value******
Italian Cohort	1,005	2.0%	0.57 (0.53-0.60)	1.30 (1.14-1.49)	1.5×10^{-4}	3.4%	0.59 (0.56-0.63)	1.43 (1.24-1.64)	6.8×10^{-7}
French Cohort	859	1.8%	0.57 (0.53-0.61)	1.27 (1.10-1.45)	7.6×10^{-4}	2.8%	0.58 (0.55-0.62)	1.36 (1.18-1.57)	2.6×10^{-5}
German Cohort	571	2.3%	0.58 (0.53-0.63)	1.35 (1.12-1.62)	1.9×10^{-3}	4.4%	0.60 (0.56-0.65)	1.54 (1.26-1.88)	2.0×10^{-5}
Czech Cohort	402	1.7%	0.57 (0.51-0.63)	1.23 (1.03-1.46)	2.4×10^{-2}	2.0%	0.57 (0.52-0.63)	1.23 (1.04-1.45)	1.5×10^{-2}
Hungarian Cohort	393	2.8%	0.59 (0.53-0.65)	1.40 (1.10-1.79)	5.7×10^{-3}	4.4%	0.61 (0.55-0.67)	1.54 (1.21-1.96)	5.1×10^{-4}
Chinese Cohort	595	1.6%	0.57 (0.52-0.62)	1.29 (1.07-1.57)	8.0×10^{-3}	2.7%	0.59 (0.54-0.63)	1.36 (1.14-1.62)	6.2×10^{-4}
Japanese Cohort	512	2.7%	0.59 (0.54-0.64)	1.34 (1.12-1.60)	1.5×10^{-3}	4.0%	0.61 (0.56-0.65)	1.38 (1.17-1.62)	1.2×10^{-4}
African-American Cohort	85	4.6%	0.63 (0.50-0.76)	1.50 (0.93-2.41)	9.6×10^{-2}	5.1%	0.64 (0.51-0.77)	1.63 (0.94-2.82)	8.1×10^{-2}
**All Replication Cohorts**	**4,422**	**2.2%**	**0.58 (0.56-0.59)**	**1.29 (1.22-1.37)**	**5.4×10^{-17}**	**3.2%**	**0.59 (0.57-0.61)**	**1.36 (1.28-1.45)**	**3.3×10^{-24}**
GWAS Discovery	2,091	7.0%	0.63 (0.60-0.65)	1.70 (1.54-1.88)	1.9×10^{-24}	7.6%	0.64 (0.61-0.66)	1.69 (1.54-1.86)	1.5×10^{-26}
GWAS Asian Follow-up	1,384	5.3%	0.61 (0.58-0.64)	1.65 (1.44-1.89)	5.8×10^{-13}	5.0%	0.61 (0.58-0.64)	1.57 (1.39-1.78)	1.2×10^{-12}
GWAS European Follow-up	2,156	4.3%	0.60 (0.58-0.63)	1.46 (1.34-1.60)	1.6×10^{-16}	5.3%	0.61 (0.59-0.64)	1.56 (1.42-1.71)	1.0×10^{-19}
**All GWAS Cohorts**	**5,631**	**5.0%**	**0.61 (0.60-0.62)**	**1.51(1.43-1.60)**	**3.1×10^{-46}**	**5.7%**	**0.62 (0.60-0.63)**	**1.56 (1.47-1.65)**	**4.1×10^{-52}**
All Asian Cohorts Combined	4,582	4.5%	0.60 (0.59-0.62)	1.53 (1.43-1.64)	3.0×10^{-34}	5.0%	0.61 (0.59-0.63)	1.52 (1.43-1.62)	2.6×10^{-38}
All European Cohorts Combined	5,386	2.6%	0.58 (0.57-0.60)	1.34 (1.26-1.41)	3.7×10^{-24}	3.6%	0.59 (0.58-0.61)	1.42 (1.34-1.51)	6.7×10^{-33}
**All Cohorts Combined**	**10,053**	**3.8%**	**0.60 (0.59-0.61)**	**1.42 (1.36-1.50)**	**6.2×10^{-63}**	**4.7%**	**0.61 (0.60-0.62)**	**1.47 (1.42-1.54)**	**1.2×10^{-76}**

# Number of analyzed individuals with 100% non-missing genotypes across all 7 scored loci.

* R^2: Nagelkerke R square expressed as percent

** C-statistic: area under the ROC curve and its 95% confidence interval.

*** odds ratio per one standard deviation of the standardized risk score and its 95% confidence interval.

**** Wald's test for risk score as a quantitative predictor of disease status.
