# Supplementary material for: Geographic Differences in Genetic Susceptibility to IgA Nephropathy: GWAS Replication Study and Geospatial Risk Analysis
Source: PLoS Genet. 2012 Jun 21;8(6):e1002765. doi: 10.1371/journal.pgen.1002765 (PMC3380840; doi:10.1371/journal.pgen.1002765)
Supplement: Table S8 — African, Middle Eastern, and European populations included in the geospatial risk analysis. The populations were grouped by their continental origin and sorted based on the median genetic risk score. (PDF) [file pgen.1002765.s011.pdf]

Supplemental Table 8. African, Middle Eastern, and European populations included in the geospatial risk analysis. The populations were grouped by their continental origin and sorted based on the median genetic risk score.

Continental Group	Country/Region	Population/Site	Sample Source	Sample Size	Longitude	Latitude	Median Standardized Risk Score
Africa	Central African Republic	Biaka Pygmies	HGDP	32	17.0	4.0	-0.77
Africa	Algeria Mzab	Mozabite	HGDP	30	3.0	32.0	-0.64
Africa	South Africa	Bantu	HGDP	8	25.2	-25.8	-0.62
Africa	Namibia	San	HGDP	6	20.0	-21.0	-0.55
Africa	Nigeria	Yoruba	HGDP	24	6.0	8.0	-0.41
Africa	Nigeria	YRI	HAPMAP3	112	3.9	7.4	-0.39
Africa	Senegal	Mandenka	HGDP	24	-12.0	12.0	-0.29
Africa	Congo	Mbuti Pygmies	HGDP	15	29.0	1.0	-0.27
Africa	Kenya Kinyawa	MKK	HAPMAP3	140	35.2	-1.5	-0.25
Africa	Kenya Webuye	LWK	HAPMAP3	89	34.8	0.6	-0.14
Africa	Kenya	Bantu	HGDP	12	37.0	-3.0	0.13
Middle East	Israel Negev	Bedouin	HGDP	48	35.0	31.0	-0.60
Middle East	Israel Central	Palestinian	HGDP	51	35.0	32.0	-0.13
Middle East	Lebanon Beirut	Arab	Present Study	29	35.5	33.9	0.12
Middle East	Israel Carmel	Druze	HGDP	46	35.0	32.0	0.31
Europe	North Italy	Italian, Bergamo	HGDP	13	10.0	46.0	-0.33
Europe	Sardinia	Sardinian	HGDP	28	9.0	40.0	-0.33
Europe	Russia Caucasus	Adygei	HGDP	17	39.0	44.0	-0.33
Europe	Czech Republic	Czech, Prague	Present Study	187	14.5	50.1	-0.27
Europe	North Italy	Italian, Lecco	IgAN GWAS	24	9.4	45.9	-0.26
Europe	North Italy	Italian, Firenze	HGDP	8	11.0	43.0	-0.18
Europe	France	French, St.Etienne	Present Study	230	1.2	43.6	-0.16
Europe	France	French. Basque	HGDP	24	0.0	43.0	-0.12
Europe	North Italy	Italian, Trento	Present Study	27	11.1	46.1	-0.12
Europe	North Italy	Italian,TSI	HAPMAP3	86	11.0	43.6	-0.09
Europe	France	French	HGDP	29	2.0	46.0	-0.08
Europe	Germany	German, Hamburg-Eppendorf	Present Study	74	10.0	53.6	0.00
Europe	North Italy	Italian, Firenze	Present Study	34	11.0	43.0	0.01
Europe	Germany	German, Aachen	Present Study	268	6.1	50.8	0.03
Europe	South Italy	Italian, Foggia	Present Study	146	15.6	41.4	0.03
Europe	Hungary	Hungarian, Pecs	Present Study	268	18.3	46.0	0.03
Europe	North Italy	Italian, Genoa	Present Study	264	8.9	44.4	0.10
Europe	North Italy	Italian, Bologna	Present Study	26	11.4	44.5	0.12
Europe	North Italy	Italian, Modena	Present Study	35	10.9	44.5	0.12
Europe	North Italy	Italian, Torino	Present Study	277	7.7	45.1	0.12
Europe	North Italy	Italian, Trieste	Present Study	89	13.6	45.9	0.15
Europe	France	French, Paris	Present Study	159	2.2	48.5	0.16
Europe	North Italy	Italian, Brescia	Present Study	25	10.2	45.5	0.19
Europe	North Italy	Italian, Brescia	IgAN GWAS	152	10.2	45.5	0.24
Europe	North Italy	Italian, Cremona	IgAN GWAS	33	10.0	45.1	0.24
Europe	Poland	Polish, Warsaw	HyperGENES	94	21.0	52.2	0.24
Europe	Scotland	Orkney Islands	HGDP	16	-3.0	59.0	0.30
Europe	North Italy	Italian, Valtrompia	Present Study	51	10.2	45.7	0.31
Europe	North Western Russia	Russian	HGDP	25	40.0	61.0	0.47
