# Supplementary material for: Geographic Differences in Genetic Susceptibility to IgA Nephropathy: GWAS Replication Study and Geospatial Risk Analysis
Source: PLoS Genet. 2012 Jun 21;8(6):e1002765. doi: 10.1371/journal.pgen.1002765 (PMC3380840; doi:10.1371/journal.pgen.1002765)
Supplement: Table S9 — Asian, Oceanian, and American populations included in the geospatial risk analysis. The populations were grouped by their continental origin and sorted based on the median genetic risk score. (PDF) [file pgen.1002765.s012.pdf]

Supplemental Table 9. Asian, Oceanian, and American populations included in the geospatial risk analysis. The populations were grouped by their continental origin and sorted based on the median genetic risk score.

Continental Group	Country/Region	Population/Site	Sample Source	Sample Size	Longitude	Latitude	Median Standardized Risk Score
Central South Asia	Pakistan	Sindhi	HGDP	25	69.0	25.5	-0.77
Central South Asia	Pakistan	Balochi	HGDP	25	66.5	30.5	-0.60
Central South Asia	Pakistan	Brahui	HGDP	25	66.5	30.5	-0.60
Central South Asia	India	GIH	HAPMAP3	88	71.2	22.3	-0.56
Central South Asia	Pakistan	Burusho	HGDP	25	74.0	36.5	-0.55
Central South Asia	Pakistan	Makrani	HGDP	25	64.0	26.0	-0.51
Central South Asia	Pakistan	Pathan	HGDP	23	70.5	33.5	-0.50
Central South Asia	Pakistan	Kalash	HGDP	25	71.5	36.0	-0.31
Central South Asia	China	Uygur	HGDP	10	81.0	44.0	-0.11
Central South Asia	Pakistan	Hazara	HGDP	24	70.0	33.5	-0.06
East Asia	China	Tu	HGDP	10	101.0	36.0	-0.33
East Asia	China	Daur	HGDP	9	124.0	48.5	-0.06
East Asia	China	Mongola	HGDP	10	111.0	45.0	0.06
East Asia	China	Yizu	HGDP	10	103.0	28.0	0.30
East Asia	China	Han	IgAN GWAS	900	116.4	39.9	0.50
East Asia	China	Dai	HGDP	10	100.0	21.0	0.52
East Asia	China	Miaozu	HGDP	10	109.0	28.0	0.54
East Asia	China	Xibo	HGDP	9	81.5	43.5	0.57
East Asia	Siberia	Yakut	HGDP	25	129.5	63.0	0.61
East Asia	China	Han	Present Study	266	116.4	39.9	0.62
East Asia	China	CHB	HAPMAP3	81	116.4	39.9	0.62
East Asia	Japan	JPT	HAPMAP3	86	139.7	35.7	0.62
East Asia	China	Han	HGDP	44	114.0	37.5	0.64
East Asia	China	Hezhen	HGDP	9	133.5	47.5	0.64
East Asia	China	Shanghai	IgAN GWAS	711	121.5	31.2	0.73
East Asia	Japan	Niigata	Present Study	267	139.1	37.9	0.73
East Asia	Japan	Japanese	HGDP	29	138.0	38.0	0.83
East Asia	China	She	HGDP	10	119.0	27.0	0.88
East Asia	Cambodia	Cambodians	HGDP	11	105.0	12.0	0.89
East Asia	China	Lahu	HGDP	10	100.0	22.0	0.89
East Asia	China	Tujia	HGDP	10	109.0	29.0	1.08
East Asia	China	Oroqen	HGDP	10	126.5	50.5	1.26
East Asia	China	Naxi	HGDP	9	100.0	26.0	1.58
Oceania	New Guinea	Papuan	HGDP	17	143.0	-4.0	-0.38
Oceania	Bougainville	NAN-Melanesian	HGDP	19	155.0	-6.0	0.64
America	USA	MEX	HAPMAP3	48	-118.2	34.1	0.40
America	Mexico	Pima	HGDP	25	-108.0	29.0	0.57
America	Colombia	Colombians	HGDP	13	-68.0	3.0	0.57
America	Mexico	Maya	HGDP	25	-91.0	19.0	0.64
America	Brazil	Surui	HGDP	21	-62.0	-11.0	0.64
America	Brazil	Karitiana	HGDP	24	-63.0	-10.0	0.89
