# Supplementary material for: Geographic Differences in Genetic Susceptibility to IgA Nephropathy: GWAS Replication Study and Geospatial Risk Analysis
Source: PLoS Genet. 2012 Jun 21;8(6):e1002765. doi: 10.1371/journal.pgen.1002765 (PMC3380840; doi:10.1371/journal.pgen.1002765)
Supplement: Table S10 — Prevalence and Incidence of ESRD due to IgAN in Europe. Primary data obtained from the ERA-EDTA Registry. (PDF) [file pgen.1002765.s013.pdf]

Supplemental Table 10. Prevalence and Incidence of ESRD due to IgAN in Europe. Primary data obtained from the ERA-EDTA Registry.

Country	Yearly Incidence (PMP) #	Population* (1000s)	ERA-EDTA Prevalence Data (December 31, 2009)					
			Prevalent Cases (Counts)			IgAN Prevalence		
			ESRD	Primary GN	IgAN	% ESRD	% Primary GN	PMP ##
**Greece**	2.5	11,283	12,018	2,319	230	1.9	9.9	19.6
**Spain**	3.2	35,423	37,349	8,142	1,539	4.1	18.9	45.5
**Italy**	2.7	29,675	33,569	7,016	1,162	3.5	16.6	36.3
**France**	5.6	36,819	38,387	8,560	2,477	6.5	28.9	68.4
**Austria**	3.5	8,355	8,177	1,954	323	4.0	16.5	37.9
**Belgium ^**	3.6	10,760	12,558	2,286	443	3.5	19.4	40.3
**The Netherlands**	2.4	16,530	14,794	2,559	628	4.2	24.5	36.7
**UK ^**	4.2	61,791	50,513	9,294	2,718	5.4	29.2	44.8
**Denmark**	1.6	5,579	4,677	940	94	2.0	10.0	16.9
**Sweden**	5.4	9,299	8,277	2,160	732	8.8	33.9	78.3
**Norway**	5.7	4,829	4,066	1,212	246	6.1	20.3	51.5
**Finland**	5.0	5,339	4,166	904	336	8.1	37.2	60.3
**Iceland**	5.3	318	172	40	17	9.9	42.4	60.2

* Based on the 2009 ERA-EDTA report.
# Incident ESRD cases due to IgAN per million population (PMP) based on a 3-year period (2007, 2008, and 2009).
Prevalent ESRD cases due to IgAN per million population (PMP) for 2009.
^ Patients younger than 20 years of age are not reported.
Data for France is based on the regions Alsace, Auvergne, Bourgogne, Bretagne, Champagne-Ardenne, Corse, Haute Normandie, Languedoc Roussillon, Limousin, Midi-Pyrénées, Pays de Loire, Picardie, Poitou-Charentes, Provence-Alpes-Côte d Azur, Rhône Alpes, and the overseas department of Reunion.
Data for Italy is based on the regions Abruzzi, Apulia, Basilicata, Calabria, Emilia-Romagna, Friuli-Venezia Giulia, Lombardy, Marche, Sardinia, Tuscany, Umbria, and Veneto.
Data for Spain is based on the regions Andalusia, Asturias, Basque country, Cantabria, Castile and Leon, Castile-La Mancha, Catalonia, Extremadura, Galicia and Valencian region.
