# Supplementary material for: Geographic Differences in Genetic Susceptibility to IgA Nephropathy: GWAS Replication Study and Geospatial Risk Analysis
Source: PLoS Genet. 2012 Jun 21;8(6):e1002765. doi: 10.1371/journal.pgen.1002765 (PMC3380840; doi:10.1371/journal.pgen.1002765)
Supplement: Table S11 — Prevalence and Incidence of ESRD due to IgAN in the US. Primary data obtained from the USRDS Annual Report, 2011. (PDF) [file pgen.1002765.s014.pdf]

Supplemental Table 11. Prevalence and Incidence of ESRD due to IgAN in the US. Primary data obtained from the USRDS Annual Report, 2011.

			USRDS Prevalence Data (December 31, 2009)					
			Prevalent Cases (Counts)			IgAN Prevalence		
**Ethnicity**	**Yearly Incidence (PMP) #**	**Population (1000s)***	**ESRD**	**Primary GN**	**IgAN**	**% ESRD**	**% Primary GN**	**PMP ##**
**US African-American**	1.5	39,641	180,567	21,192	598	0.33	2.82	14.4
**US White-American**	2.8	244,298	347,420	52,164	6,660	1.92	12.77	26.4
**US Native-Americans**	5.5	3,151	7,428	978	181	2.44	18.53	50.7
**US Asian-American**	10.8	14,014	30,285	6,357	1,522	5.03	23.94	93.2

* Based on the 2009 US Census Data
# Incident ESRD cases due to IgAN per million population (PMP) based on a 5-year period (2005-2009).
Prevalent ESRD cases due to IgAN per million population (PMP) for 2009.
